# Sexual selection and the evolution of male pheromone glands in philanthine wasps (Hymenoptera, Crabronidae)

**DOI:** 10.1186/s12862-017-0963-6

**Published:** 2017-06-06

**Authors:** Katharina Weiss, Gudrun Herzner, Erhard Strohm

**Affiliations:** Evolutionary Ecology Group, Institute of Zoology, University of Regensburg, Universitätsstr. 31, 93053 Regensburg, Germany

**Keywords:** Philanthinae, Beewolves, Sexual selection, Interspecific variation, Postpharyngeal gland, Mandibular gland, Comparative morphology, Categorical principal components analysis, Ancestral state reconstruction

## Abstract

**Background:**

Sexual selection is thought to promote evolutionary changes and diversification. However, the impact of sexual selection in relation to other selective forces is difficult to evaluate. Male digger wasps of the tribe Philanthini (Hymenoptera, Philanthinae) scent mark territories to attract receptive females. Consequently, the organs for production and storage of the marking secretion, the mandibular gland (MG) and the postpharyngeal gland (PPG), are subject to sexual selection. In female Philanthini, these glands are most likely solely subject to natural selection and show very little morphological diversity. According to the hypothesis that sexual selection drives interspecific diversity, we predicted that the MG and PPG show higher interspecific variation in males than in females. Using histological methods, 3D-reconstructions, and multivariate statistical analysis of morphological characters, we conducted a comparative analysis of the MG and the PPG in males of 30 species of Philanthini and three species of the Cercerini and Aphilanthopsini, two related tribes within the Philanthinae.

**Results:**

We found substantial interspecific diversity in gland morphology with regard to gland incidence, size, shape and the type of associated secretory cells. Overall there was a phylogenetic trend: Ensuing from the large MGs and small PPGs of male Cercerini and Aphilanthopsini, the size and complexity of the MG was reduced in male Philanthini, while their PPG became considerably enlarged, substantially more complex, and associated with an apparently novel type of secretory cells. In some clades of the Philanthini the MG was even lost and entirely replaced by the PPG. However, several species showed reversals of and exceptions from this trend. Head gland morphology was significantly more diverse among male than among female Philanthinae.

**Conclusion:**

Our results show considerable variation in male head glands including the loss of an entire gland system and the evolution of a novel kind of secretory cells, confirming the prediction that interspecific diversity in head gland morphology is higher in male than in female Philanthini. We discuss possible causes for the remarkable evolutionary changes in males and we conclude that this high diversity has been caused by sexual selection.

**Electronic supplementary material:**

The online version of this article (doi:10.1186/s12862-017-0963-6) contains supplementary material, which is available to authorized users.

## Background

Ever since Charles Darwin introduced sexual selection as a distinct evolutionary force [1, 2], its importance relative to other evolutionary processes has been debated [3–10]. In particular, the potential of sexual selection as a driving force for speciation has received much attention [11–15]. Generally, sexual selection is assumed to promote rapid evolutionary change and population divergence ([8, 16–22], but see e.g. [3, 15]) due to different mechanisms like the Fisher-Zahavi processes [23–25] and sexual antagonism [26]. However, as outlined by Panhuis et al. [14], observed diversity in a trait presumably under sexual selection may also have been caused by other evolutionary forces like natural selection, genetic drift, or mutation. Hence, one major problem in the study of sexual selection is the assessment of its effect relative to other potential causes of evolutionary change [14, 27].

Whereas the evolution of visual and acoustic courtship signals and their structural basis have been studied extensively (e.g. [28, 29]), the glands involved in the production of sex pheromones have received comparatively little attention [30] although chemical communication is probably the oldest and predominant mode of communication in most animal taxa [31]. Here we test the hypothesis that head glands of male digger wasps that are subject to sexual selection show higher interspecific diversity than the same glands in females, where they are under natural selection.

The mandibular glands (MG) and the postpharyngeal glands (PPG) of the solitary digger wasp subfamily Philanthinae (Hymenoptera, Crabronidae) are an excellent model system to study the relative contribution of sexual selection to evolutionary change since these glands occur in both sexes but are subject to different selection regimes in males and females. The Philanthinae consist of eight genera, separated into three tribes [32]: the Cercerini (comprising the three genera (*Cerceris* + *Eucerceris*) + *Pseudoscolia*), the Aphilanthopsini (comprising *Clypeadon* and *Aphilanthops*), and the Philanthini, the so-called beewolves (comprising (*Philanthus* + *Trachypus*) + *Philanthinus,* with *Trachypus* most probably being a subgenus of *Philanthus* [32, 33]). The members of the subfamily largely share basic life-history characters, in particular with regard to female nesting behavior (e.g. [34–40]) and male reproductive behavior (e.g. [37, 41–47]).

As best documented for the genus *Philanthus*, males establish small territories in the vicinity of female nesting aggregations (e.g. [37, 43, 44, 47]) and scent-mark their territories with a secretion from their large head glands to attract receptive females (e.g. [37, 48–50]). Scent marking and territoriality is also known from males of some species of the tribe Cercerini [41, 42, 45, 46] and at least two species of the Aphilanthopsini [41, 46]. Earlier publications on *Philanthus* assumed that the males’ marking secretion is produced and stored in the MG (reviewed in [37]). In the European beewolf *Philanthus triangulum* the marking secretion is in fact most likely synthesized in the gland cells of the MG [51], but the main storage organ is the remarkably enlarged PPG [50, 52]. The MG and the PPG together are considerably larger than the brain. The huge size of the glands and the tremendous amounts of marking secretion that are produced and stored [49, 50] clearly illustrate the importance of these glands for beewolf males. Moreover, there is evidence that females prefer larger males that produce and store larger amounts of pheromone in their glands and apply more secretion to their territories (Strohm et al., unpublished).

In addition to the quantity of the marking secretion, its composition likely plays a decisive role for male attractiveness. In *P. triangulum*, the composition of the males’ marking secretion has presumably been influenced by a female sensory bias [53–55]. Female *P. triangulum* use (*Z*)-11-eicosen-1-ol as a kairomone to identify their only prey, honeybee workers (*Apis mellifera*), and have evolved a high sensitivity for this compound [53]. Males exploit this pre-existing female sensory bias to increase their territories’ conspicuousness to females by using (*Z*)-11-eicosen-1-ol as the major component of their marking secretion [49, 50, 53]. Taken together, these findings imply that both the amount and the composition of the marking pheromone are important determinants of male reproductive success. Consequently, the secretory cells that produce the marking secretion and the gland reservoirs that store it are subject to strong sexual selection.

Female Philanthinae also possess an MG and a PPG [56–59]. Females of this subfamily mass-provision subterranean brood cells with paralyzed insects as food for their progeny (e.g. [34, 35, 37, 60]). Since the larval provisions are prone to fungal infestation (e.g. [61]), at least some species of the Philanthini have evolved an intriguing defense mechanism that involves the PPG. Females literally embalm their prey with the secretion of the PPG [58, 59, 62–64]. This embalming reduces moisture on the prey’s cuticle and hence delays fungal growth [61, 63, 65]. Since all Philanthini appear to face similar challenges regarding fungal infestation of larval provisions, their PPGs can be expected to be subject to similar natural selection pressures. Even though nothing is known about the function of the female MG, it is most likely also subject to natural, rather than sexual selection. The morphology of the PPG and MG has been shown to be rather uniform among female Philanthini [59].

Based on the hypothesis that sexual selection causes greater interspecific diversity than natural selection (e.g. [8, 16–18, 20]), we predict that the morphology of head glands varies more among male than among female Philanthini. Other evolutionary processes like genetic drift and mutations should affect the glands of both sexes in the same way. Since detailed morphological studies on male head glands were only available for two species of the subfamily Philanthinae, *P. triangulum* (MG: [51], PPG: [52])] and *Cerceris rybyensis* (MG [56]), we conducted a comparative analysis of the PPG and MG of male Philanthinae. Using histological methods and 3D-reconstructions, we investigated males of 30 species of Philanthini, covering all major phylogenetic lineages. Moreover, we included three species of the closely related tribes Cercerini and Aphilanthopsini. Based on 14 morphological characters, comprising incidence, location, size, shape and structure of gland reservoirs, as well as histological characteristics of associated secretory cells, we performed a multivariate statistical analysis of PPGs and MGs to assess the pattern of interspecific variation in gland morphology. In order to reveal possible phylogenetic trends, we mapped gland morphology on a recent molecular phylogeny of the Philanthinae [33]. To explore the evolutionary origin and fate of important characters, we conducted ancestral state reconstruction analyses [66]. We discuss the interspecific variation in male head gland morphology and assess the role of sexual selection in the evolution of these glands in male Philanthinae. Using the variation of female head glands [59] as a reference under natural selection, we test whether head gland morphology shows higher diversity in males.

## Methods

### Study material

Overall, males of 33 species and one subspecies from five genera, representing the three tribes of the crabronid subfamily Philanthinae were examined (Table 1). We refer to the phylogeny and phylogeography of the Philanthinae according to Kaltenpoth et al. [33]. Designation of zoogeographic regions follows Holt et al. [67]. Our main focus was on the tribe Philanthini, the so-called beewolves. The Philanthini can be grouped into five clades, largely coinciding with their geographic distribution [33] and we investigated representatives of all of these clades (Table 1): One species of the basal genus *Philanthinus*, two species of a small clade of Palearctic, Indian, and Afrotropical species of the genus *Philanthus*, forming the sister group to all other *Philanthus*, ten species of a clade comprising all other Palearctic, Indian, and Afrotropical *Philanthus*, 14 Nearctic *Philanthus* species, and three species of the Neotropical subgenus *Trachypus*. The total number of described species is four for *Philanthinus,* 136 for *Philanthus* and 31 for *Trachypus* [68, 69]. Moreover, we included three species of the two other tribes of the Philanthinae, namely one Nearctic *Clypeadon* species (tribe Aphilanthopsini, 13 described species) and two Palearctic *Cerceris* (tribe Cercerini, 905 described species). Each species under study is assigned an ID number (Table 1) that is used throughout the manuscript and Additional files.

**Table 1:** Species included in the comparative morphological study of head glands of male Philanthinae

Tribe	ID	Species	N	Country	3D
Cercerini	1	*Cerceris quinquefasciata*	2	Germany	yes
	2	*Cerceris rybyensis*	2	Germany	yes
Aphilanthopsini	3	*Clypeadon laticinctus*	5	USA	yes
Philanthini	4	*Philanthinus quattuordecimpunctatus*	3	Turkey	yes
	5	*Philanthus* cf. *basalis*	1	India	no
	6	*Philanthus pulcherrimus*	1	India	yes
	7	*Philanthus spec* (India)	1	India	yes
	8	*Philanthus venustus*	2	Turkey	yes
	9	*Philanthus capensis*	1	South Africa	yes
	10	*Philanthus coronatus*	2	Germany	yes
	11	*Philanthus fuscipennis*	1	South Africa	yes
	12	*Philanthus histrio*	2	South Africa	no
	13	*Philanthus loefflingi*	3	South Africa	yes
	14	*Philanthus melanderi*	1	South Africa	yes
	15	*Philanthus rugosus*	3	South Africa	yes
	16	*Philanthus triangulum triangulum*	3	Germany	no
	17	*Philanthus triangulum diadema*	3	South Africa	yes
	18	*Philanthus albopilosus*	2	USA	yes
	19	*Philanthus barbiger*	3	USA	yes
	20	*Philanthus bicinctus*	2	USA	yes
	21	*Philanthus crotoniphilus*	2	USA	yes
	22	*Philanthus gibbosus*	3	USA	no
	23	*Philanthus gloriosus*	2	USA	yes
	24	*Philanthus multimaculatus*	2	USA	yes
	25	*Philanthus occidentalis*	2	USA	no
	26	*Philanthus pacificus*	1	USA	yes
	27	*Philanthus parkeri*	1	USA	yes
	28	*Philanthus politus*	2	USA	yes
	29	*Philanthus psyche*	1	USA	yes
	30	*Philanthus pulcher*	1	USA	yes
	31	*Philanthus ventilabris*	1	USA	yes
	32	*Trachypus elongatus*	2	Brazil	yes
	33	*Trachypus flavidus*	2	Brazil	no
	34	*Trachypus patagonensis*	1	Brazil	no

Tribe: phylogenetic affiliation of the species, ID: identification number of the species, Species: Species name, N: number of specimens examined, Country: collection site of the species, 3D: 3D-reconstruction for this species conducted (yes) or not (no).

### Histology

Wasps were caught in the field in their territories or at flowers. They were cold anesthetized, decapitated and heads were fixed either in formalin-ethanol-acetic acid, alcoholic Bouin, or, in four cases, 100% ethanol [70]. After fixation, heads were rinsed, dehydrated in a graded ethanol series and propylene oxide, and embedded in Epon 812 (Polysciences Europe GmbH, Eppelheim, Germany). To facilitate the infiltration of the embedding medium into large heads, lateral parts of both compound eyes were cut off after fixation. Continuous series of sagittal semithin sections (4 μm) were cut with a microtome (Reichert Ultracut; Leica Microsystems AG, Wetzlar, Germany) equipped with a diamond knife and a large trough, mounted on microscope slides, and stained with toluidine blue [70]. The resulting series of histological sections were investigated by light microscopy (bright field, differential interference contrast, and phase contrast; Zeiss Axiophot 2; Carl Zeiss Microscopy GmbH, Oberkochen, Germany; Leica DMLS, Leica GmbH, Wetzlar, Germany).

Designation of glands was done according to the site of their openings. Reservoirs opening near the base of the mandibles were regarded as MGs and reservoirs opening to the pharynx just proximal to the hypopharyngeal plate were regarded as PPGs. Secretory cells associated with the gland reservoirs were classified according to Noirot and Quennedey [71] whenever possible; such cells will be referred to as ‘NQ-class cells’. In addition, we detected presumably secretory cells not matching the classification of Noirot and Quennedey [71]. We include these cells as morphological characters in our analysis (see Morphological characters) but will provide extensive histological and ultrastructural details elsewhere.

All species also possessed a hypopharyngeal gland. We did not include this gland in our analysis, because several aspects contradict a role in territory marking: (1) the gland seems to be involved in nutrition and digestion [72–74], (2) it does not have a reservoir, and (3) using gas chromatography and mass spectrometry, we did not find volatile components in this gland (Strohm et al., unpublished).

### 3D-reconstruction

To visualize the overall morphology of head glands and to facilitate comparison among species, 3D-reconstructions of the head glands were generated for 27 of the 34 investigated taxa (Table 1). For two *Trachypus* and five *Philanthus* species no complete series of sections were available (Table 1); however, also for these species the available histological sections were sufficient to allow for the determination of most gland characters (see Morphological characters). Due to deficient quality of a part of the sections, reconstruction was only possible for one side of the head for *Philanthus capensis* (ID 9)*, Philanthus gloriosus* (ID 23), and *Philanthus multimaculatus* (ID 24). For 3D-reconstruction, continuous series of semithin sections of one individual per species (on average 560 sections per head; 14,980 sections in total) were photographed using a digital microscope camera (Olympus DP20; Olympus, Hamburg, Germany) attached to a light microscope (Zeiss Axiophot 2) using 2.5× or 5× PlanNeofluar objectives. The digital images were automatically aligned to each other using the software TrakEM2 [75] for the image processing software Fiji [76]; all alignments were checked and manually corrected if necessary. The outer margin of the epithelium surrounding the reservoirs of the MG and the PPG as well as the pharynx were then marked as 3D-objects in TrakEM2 by manually outlining them in each picture of a series. For *Philanthus rugosus* (ID 15), additionally secretory cells of the MG and the PPG as well as the brain and the ocelli were marked. Finally, 3D-reconstructions were calculated and visualized using Fiji’s 3D-viewer plug-in [77].

### Statistical analysis of gland morphology

#### Morphological characters

Based on an extensive examination of both semithin histological sections and 3D-reconstructions, we defined 14 morphological characters of the PPG and MG for a comparative statistical analysis of the head glands of male Philanthinae. These characters comprise information on the incidence, relative size, structure and overall shape of the glands, their location within the head capsule, as well as the type and arrangement of associated gland cells. Character states were categorized and numerically coded for statistical analysis. Due to partial deficiencies in the histological sections not all character states could be determined for all species. Detailed descriptions of the characters and character states are given in section 1 of the Additional file 1. In brief, the defined characters were: (1) ‘Overall structure of the PPG’, (2) ‘Size of the PPG relative to the head capsule’, (3) ‘Modifications of PPG morphology’, (4) ‘Branching of the PPG’, (5) ‘Numbers of openings of the lower part of the PPG to the pharynx’, (6) ‘Structure of the inner walls of the PPG’, (7) ‘Type of gland cells associated with the PPG’, (8) ‘Presence of the MG’, (9) ‘Overall structure of the MG’, (10) ‘Size of the MG relative to the head capsule’, (11) ‘Location of the MG in the head capsule’, (12) ‘Branching of the MG’, (13) ‘Structure of the inner walls of the MG’, (14) ‘Type of gland cells associated with the MG’. While the volume of a gland may vary due to differences in filling status, the longitudinal extension within the head capsule that we used as a measure of gland size is only slightly affected. If several specimens were available for a species, these had very similar morphology and did not differ with regard to the character states.

#### Data matrix for statistical analysis

The pronounced variation among species (see Results) required the differentiation of many character states. Since only a limited number of species could be analyzed there was only a low number of cases for some character states (see Additional file 1: section 1 and Table S1). Therefore, in addition to a dataset comprising all differences observed among species (‘full dataset’, Table S1), we created a second dataset, in which we pooled character states wherever reasonable (‘combined dataset’, Additional file 1: Table S2) and that we used for statistical analyses.

#### Categorical principal components analysis

To reveal patterns of character distribution among species, a categorical principal component analysis (CATPCA) was conducted using the program ‘CATPCA’ [78] implemented in the SPSS Categories module (SPSS version 21.0, IBM; Chicago, IL, USA). Two species were excluded from the CATPCA: For the Neotropical *Trachypus patagonensis* (ID 34) the insufficient quality of the single available series of histological sections only allowed to obtain reliable data on MG but not on PPG morphology (Table S1). Moreover, males of the Nearctic *Philanthus albopilosus* (ID 18) lacked well-developed head glands (see Results). Hence, the large difference of *P. albopilosus* to the other philanthine species would have unnecessarily lowered the quality parameters of the CATPCA solution. More details on the implementation of the CATPCA are given in section 2.1 of the Additional file 1.

To test whether there was an opposing trend between MG and PPG with regard to their size and complexity, we conducted phylogenetic generalized least squares regressions based on the molecular phylogeny of Kaltenpoth et al. [33]. As with the CATPCA, *P. albopilosus* was excluded from this analysis, as well as the Nearctic *Philanthus gibbosus* (ID 22), for which the size of the PPG reservoir could not be assessed (Additional file 1: Tables S1 and S2). Moreover, since the molecular phylogeny comprised only one unidentified *Cerceris* species [33], we included only *C. rybyensis* (ID 2; omitting *Cerceris quinquefasciata*, ID 4). We used the package ‘ape’ [79] in R (Version 3.3.3, [80]) to test for a correlation between MG and PPG size and MG and PPG complexity with correction for phylogenetic relationships (for more details see section 2.2, Additional file 1).

#### Hierarchical cluster analysis and phylogenetic trends in gland morphology

We tested for phylogenetic trends in gland morphology using a cophylogenetic analysis between a morphology-based dendrogram resulting from a hierarchical cluster analysis (HCA) and a molecular phylogeny [33]. The HCA was based on 13 of the 14 gland characters (see section 2.2, Additional file 1) and was conducted in PAST (Version 2.08b, [81]) with the Bray-Curtis-index as a measure of dissimilarity and ‘unweighted pair-group averages’ as clustering algorithm; the number of bootstrap replicates was set to 10,000. Cophylogenetic analyses are mostly employed to test for coevolution of parasites and their hosts. Treating the morphology-based dendrogram as ‘parasite tree’ and the molecular phylogeny of the Philanthinae [33] as ‘host tree’, the congruence between the two was tested for statistical significance using the software tool Jane 4 [82]. Details on the implementation of the HCA and the cophylogenetic analysis are given in sections 2.3 and 2.4 of the Additional file 1.

#### Ancestral state reconstructions

Our investigations revealed that two major aspects of the head glands of male Philanthinae, the MG as well as the presumed secretory cells of the PPG, showed a complex phylogenetic distribution including losses and regains (see Results). Based on the molecular phylogeny [33], we conducted ancestral state reconstructions (ASR) [66] for the presence of both the MG (character 8, Additional file 1: Tables S1 and S2) and the secretory cells of the PPG (state 0 vs. all other states of character 7, Tables S1 and S2) using the software tool Mesquite (Version 3.04, [83]). As above, since the molecular phylogeny comprised only one unidentified *Cerceris* species [33], we conducted the ASR with only *C. rybyensis* (ID 2) and *Clypeadon laticinctus* (ID 3) as outgroup species (omitting *Cerceris quinquefasciata*, ID 4). We applied maximum likelihood (ML) approaches using asymmetrical Markov k-state 2 parameter models with the rate of change between the two character states (i.e. absence vs. presence of the MG and the secretory cells of the PPG, respectively) set to 1. Since for both traits the likelihood of gain vs. loss is not known, we tested different bias ratios for gains vs. losses ranging from 10 (i.e. gains ten times more frequent than losses) to 0.1 (i.e. losses ten times more frequent than gains).

#### Comparison of morphological diversity in males and females

To formally evaluate the hypothesis that the diversity among males is larger than among females, we compiled an aggregated matrix of gland characters of males of 32 species and females of 28 species (data for females taken from [59], Additional file 2: Table S4). Most characters are shared by both sexes and the respective character states could be simply combined. However, some characters or character states had to be recoded because they were assessed differently in the sexes or the character states were more finely differentiated in females. Based on the aggregated matrix, we conducted a CATPCA as described above to illustrate the distribution of males and females with regard to their gland morphology. To test for a difference in diversity between males and females, we calculated Shannon diversity indices among the character states of the characters that occur in both sexes (four characters that are restricted to either males or females had to be omitted) and compared these values using an exact Wilcoxon matched pair test. For more details see Additional file 2.

## Results

### General aspects of gland morphology

**Fig. 1:**
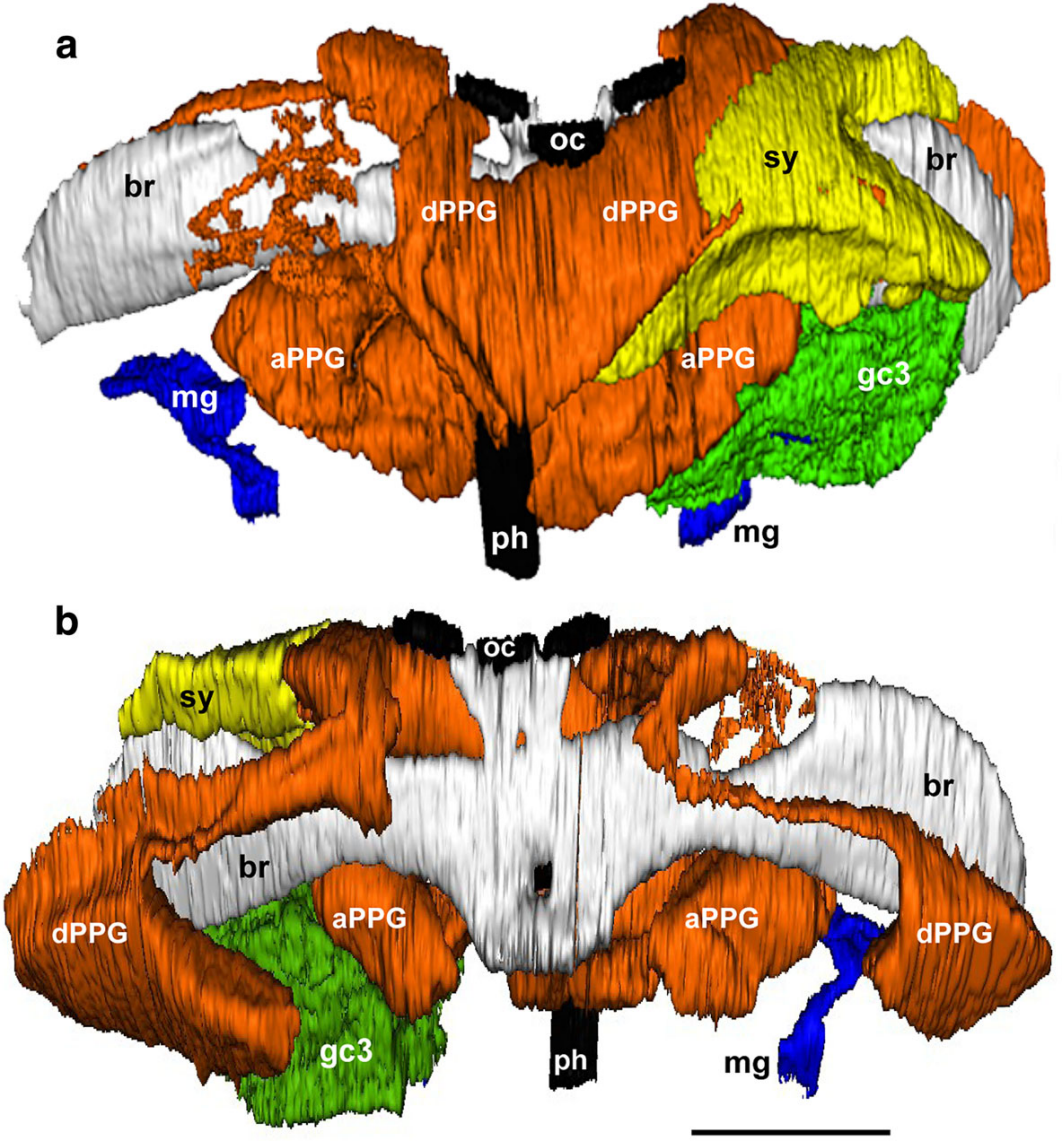
3D-reconstruction of the internal structures of a male *Philanthus rugosus* head. **a** Anterior view, **b** posterior view. The upper postpharyngeal gland reservoir (PPG; *orange*) originates dorsally from the pharynx (*black*) and basically consists of two pairs of lateral evaginations, one extending dorsally around the brain (*light grey*) (dPPG; see also Additional file 1: Figure S1 A) and one extending laterally anterior to the brain (aPPG; see also Additional file 1: Figure S1 B). The fine branches originating from the dorsal part of the upper PPG (see also Additional file 1: Figure S1 C) are surrounded by syncytia of secretory cells (*yellow*, shown only for the *left side* of the head). The upper mandibular gland reservoirs (MG; *blue*) have their openings at the dorsal mandibular base and extend laterally. The MG is associated with single NQ-class 3 gland cells (*green*, shown only for the *left side* of the head capsule). Abbreviations: aPPG, anterior parts of the upper PPG reservoir; br, brain; dPPG, dorsal parts of the upper PPG reservoir; gc3, single NQ-class 3 gland cells associated with the MG; mg, upper MG reservoir; oc, ocelli; ph, pharynx; sy, syncytia of secretory cells associated with the fine branches of the dorsal part of the upper PPG. Scale bar = 0.5 mm

In all species of Philanthinae under study, males possess either an MG, or a PPG, or both and, with one exception (*P. albopilosus*, ID 18), at least one of these glands occupies a considerable part of the head capsule. Nineteen of the 33 investigated species possess an MG that is located in the front part of the head capsule anterior to the brain and, depending on its size, may extend behind the brain, lateral from or subjacent to the PPG. The MG comprises paired reservoirs opening at the dorsal side of the mandible base and extending laterally and dorsally on both sides of the head capsule, in some cases even reaching behind the brain (Fig. 1, *P. rugosus*). Some species have only a lower MG reservoir opening at the ventral side of the mandible base and extending backwards. A few species possess both parts. MG reservoirs are surrounded by a monolayered epithelium that is moderately thick in most species. However, in some species with only an upper MG, the epithelium is distinctly thinner. The epithelial cells bear an apical cuticular intima that regularly forms a variety of conspicuous structures. Moreover, there is interspecific variation with regard to the types of secretory cells associated with the MG (see below).

All 33 investigated species possess a PPG, clearly identified by its connection to the pharynx anterior to the brain and posterior to the hypopharyngeal plate (Fig. 1). The PPG also shows considerable interspecific variation. In most species, the main upper part of the PPG basically consists of two pairs of lateral evaginations: one pair extending dorsally and in some species even around the brain (dPPG in Fig. 1) and a second pair located anterior to the brain and extending laterally towards the ventral rims of the compound eyes (aPPG in Fig. 1, for the delineation of the two parts see also Additional file 1: Figure S1 A and B). The anterior part may reach the compound eyes and, in some species, the base of the mandibles. In 14 of the 33 investigated species, there is an additional, smaller, lower part of the PPG consisting of an unpaired ventral evagination of the pharynx (Additional file 1: Figure S1 F). The walls of all parts of the PPG consist of a (partly very thin) monolayered epithelium with an apical cuticular intima. The epithelial cells generally bear hairs or scales that extend into the lumen of the gland.

**Fig. 2:**
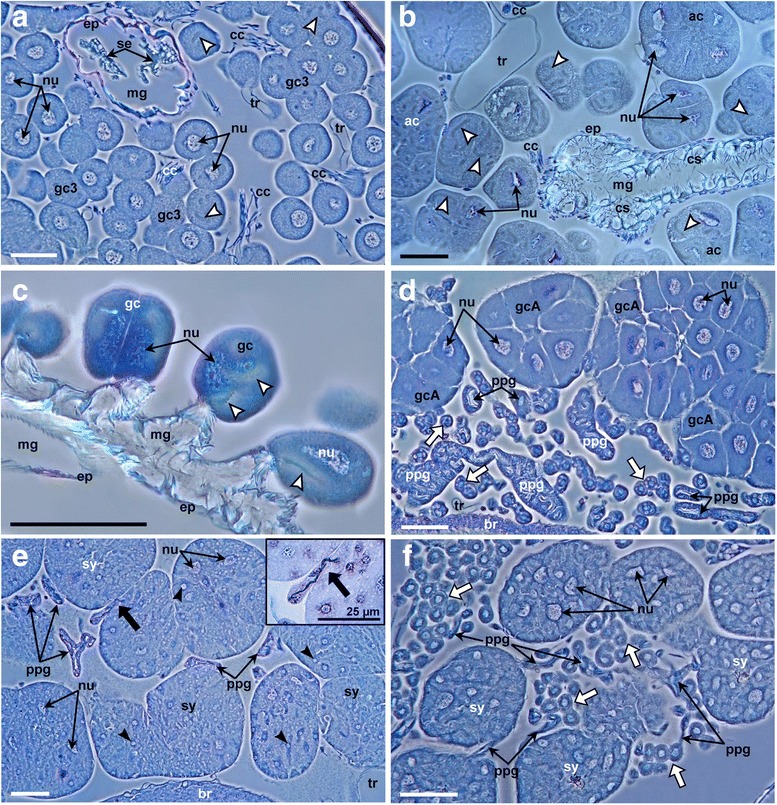
Semithin sagittal sections through the heads of male Philanthinae. **a** Single NQ-class 3 gland cells, i.e. complexes of a secretory cell and a canal cell, the latter forming a conspicuous end apparatus (*white arrow heads*) and canal that connects the secretory cell to the lumen of the MG (*Philanthus multimaculatus*, ID 24); **b** Acini of NQ-class 3 gland cells with end apparatuses (*white arrow heads*) connected to the MG reservoir by bundles of conducting canals (*Philanthus T. diadema*, ID 17); **c** Single gland cells possessing end apparatus (*white arrow heads*), thus resembling NQ-class 3 cells, but directly associated with the wall of the MG reservoir without canals (*Clypeadon laticinctus*, ID 3); **d** Aggregations of mononuclear secretory cells surrounding the fine branches of the PPG reservoir, interspersed with small rounded cells (*white arrows*) (*Philanthus venustus*, ID 8); **e** Multinuclear syncytia of secretory cells, containing many vesicles (*black arrow heads*), and in close contact to the fine branches of the PPG reservoir (*thick black arrow*; inset: detail of a PPG branch terminating in syncytium) (*Philanthus histrio*, ID 12); **f** Multinuclear syncytia of secretory cells surrounding the fine branches of the PPG reservoir and interspersed with small cells (*white arrows*) (*Philanthus crotoniphilus*, ID 21). Abbreviations: ac, acini of NQ-class 3 cells; br, brain; cc, conducting canal; cs. cuticular spines; ep, epithelium of the MG; gc. secretory cells not resembling NQ-class cells; gcA, aggregations of mononuclear secretory cells; gc3, NQ-class 3 gland cells; mg, lumen of the mandibular gland; nu, nucleus with nucleoli; ppg, fine branches of the postpharyngeal gland; se, secretion within the MG; sy, multinuclear syncytia; tr, tracheole. Scale bars [except inset in (E)] = 50 μm

The reservoirs of both glands may be associated with different types of cells (Fig. 2 and Additional file 1: Figure S2; see also Fig. 1 for the location of the cells). These cells presumably have secretory functions given their close proximity or direct contact to the reservoirs and the abundance of vesicles and nucleoli (Fig. 2). The gland cells of the MG can be differentiated into three types. In some species there are typical NQ-class 3 cells [71], i.e. complexes of a secretory cell and a canal cell, the latter forming conspicuous end apparatus and canals that connect the secretory cell to the lumen of the MG (Fig. 2a). In other species, several NQ-class 3 cells are aggregated in acini (Fig. 2b). The third type comprises secretory cells that are located directly at the wall of the reservoir and bear end apparatus but no canals (Fig. 2c). Though these cells appear to be complexes of two cells, thus resembling NQ-class 3 cells, we assign them to a different character state to account for the lack of visible canals (see also section 1, Additional file 1).

The cells associated with the PPG can occur either as aggregations of mononuclear cells (superficially resembling the acini of the MG) (Fig. 2d) or as multinuclear syncytia (Fig. 2e) (see below and Additional file 1: section 1), both showing clear signs of secretory activity: large nuclei, conspicuous nucleoli and numerous vesicles (black arrowheads in Fig. 2e). However, these cells are clearly not NQ-class 3 cells, since they lack an end apparatus and canals. Moreover, they are not part of the gland epithelium and are, thus, not NQ-class 1 cells either. Remarkably, the PPG reservoir itself is extensively ramified with the thinnest branches reaching into the cell aggregations or syncytia (black arrow and inset in Fig. 2e). In some species the cell aggregations or syncytia are interspersed with conspicuous small rounded cells with barely any cytoplasm (white arrows in Fig. 2d and f).

### Pattern of interspecific variation in gland morphology

**Fig. 3:**
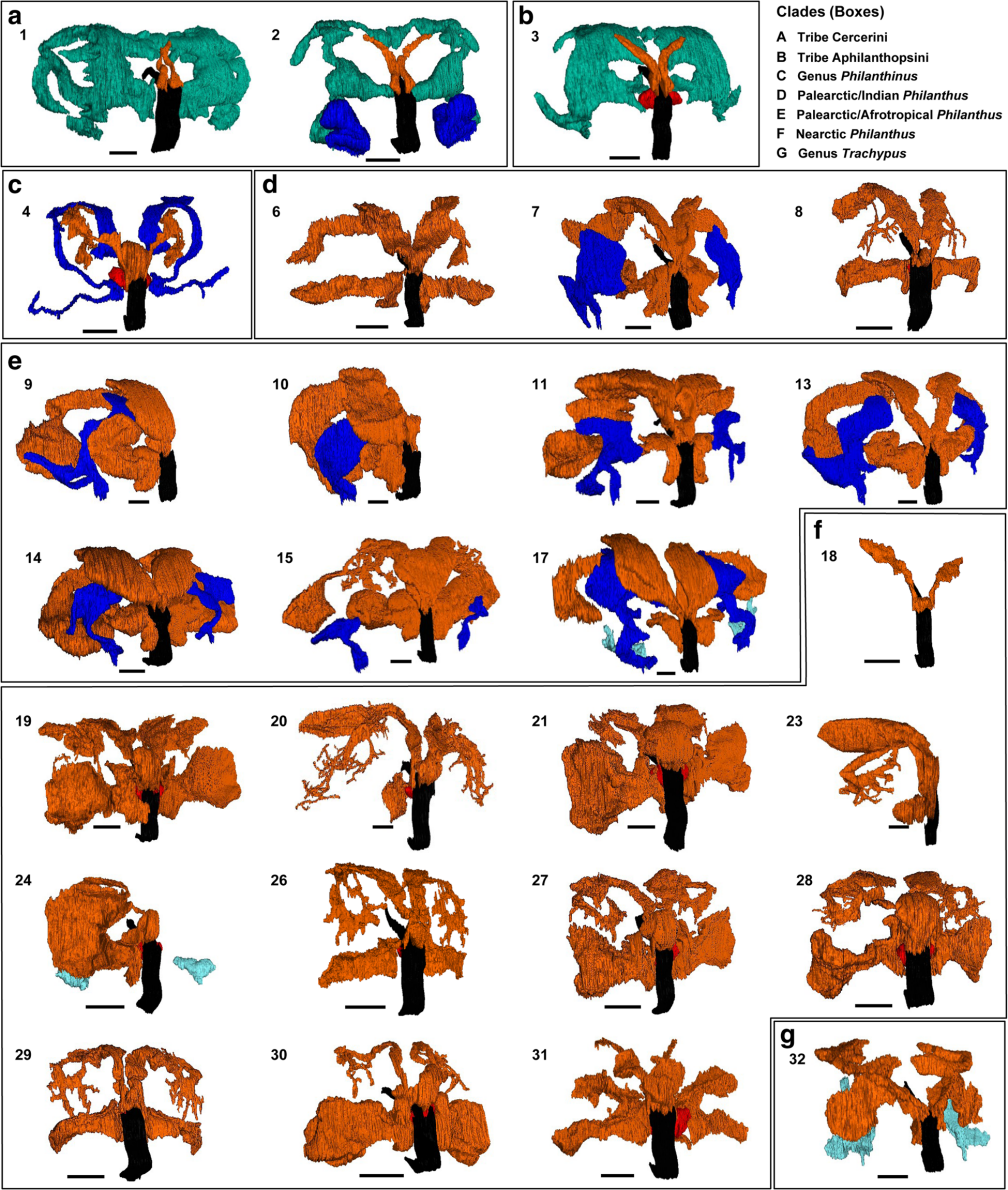
3D-reconstructions of the postpharyngeal gland (PPG) and the mandibular gland (MG) of male Philanthinae. Species IDs (corresponding to Table 1): (1) *Cerceris quinquefasciata*, (2) *Cerceris rybyensis*, (3) *Clypeadon laticinctus*, (4) *Philanthinus quattuordecimpunctatus*, (6) *Philanthus pulcherrimus*, (7) *Philanthus spec.* (India), (8) *Philanthus venustus*, (9) *Philanthus capensis*, (10) *Philanthus coronatus*, (11) *Philanthus fuscipennis*, (13) *Philanthus loefflingi*, (14) *Philanthus melanderi*, (15) *Philanthus rugosus*, (17) *Philanthus triangulum diadema*, (18) *Philanthus albopilosus*, (19) *Philanthus barbiger*, (20) *Philanthus bicinctus*, (21) *Philanthus crotoniphilus*, (23) *Philanthus gloriosus*, (24) *Philanthus multimaculatus*, (26) *Philanthus pacificus*, (27) *Philanthus parkeri*, (28) *Philanthus politus*, (29) *Philanthus psyche*, (30) *Philanthus pulcher*, (31) *Philanthus ventilabris*, (32) *Trachypus elongatus*. *Boxes* a - g indicate phylogeographic classification of species (according to [33], see key in figure). Color code for 3D-structures: *orange*, upper part of the PPG; *red*, lower part of the PPG; *dark blue*, upper part of the MG; *light blue*, lower part of the MG, turquoise, thin-walled MG reservoir of the Cercerini and Aphilanthopsini; *black*, pharynx. Due to limited availability of serial histological sections, for species (9), (10), and (23) only the right side of the paired gland reservoirs could be reconstructed, while for species (24), both reservoirs of the MG, but only the right half of the PPG are depicted; for species (6), (7), (10)-(15), and (31), the fine branches originating from the main PPG reservoir [see e.g. species (8) and (20)] could not be reconstructed based on semithin section due to their very fine structure and high number. Scale bars = 0.25 mm

**Fig. 4:**
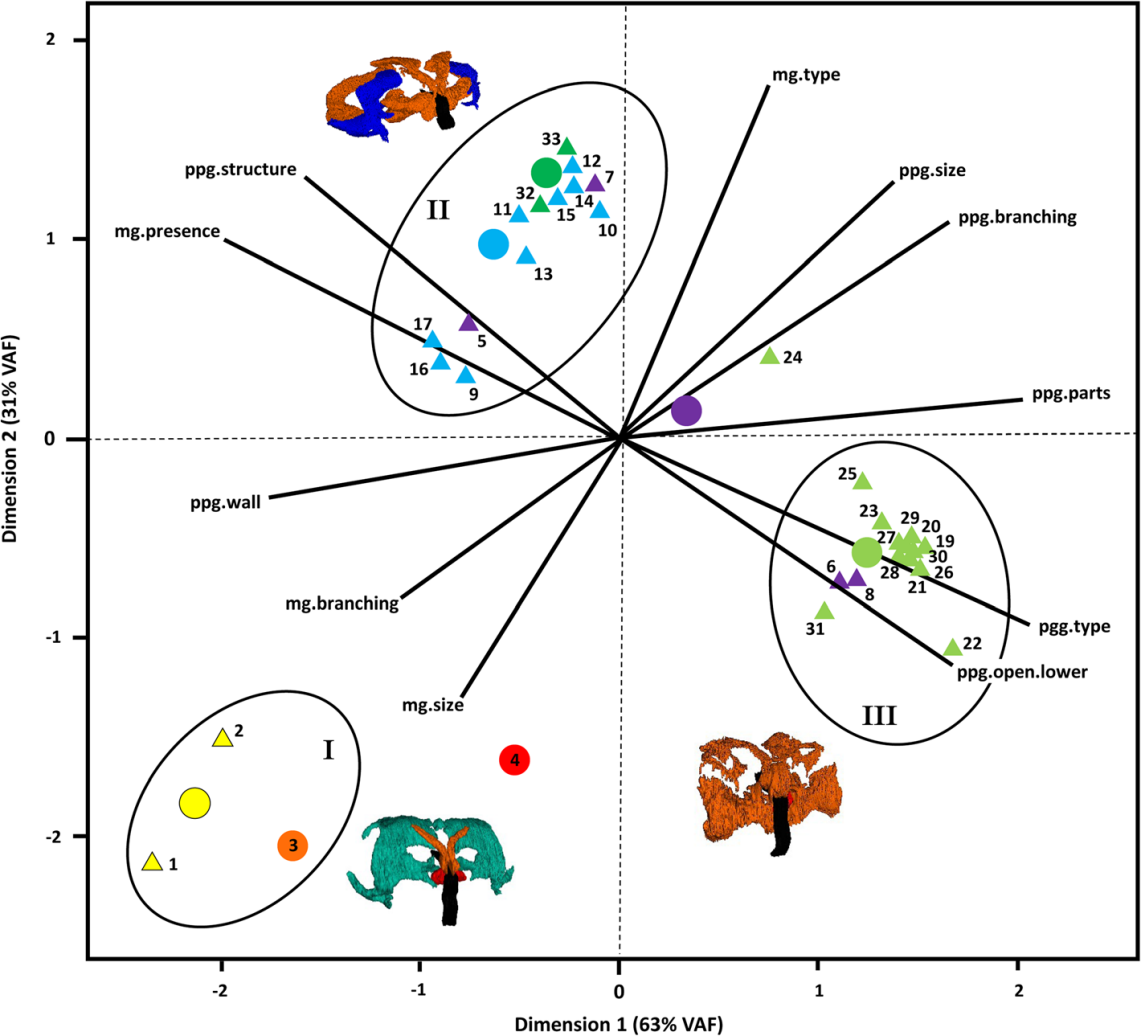
First two dimensions (VAF: percent of variance accounted for) of the CATPCA of the head gland morphology of male Philanthinae. Based on the morphology of their head glands, the species form three distinct groups (ellipses; exemplary 3D-reconstructions: (I) *Cerceris quinquefasciata*, (II) *Philanthus rugosus*, (III) *Philanthus politus*). *Triangles*: object scores of single species (IDs correspond to Table 1), *Vectors*: component loadings of morphological characters, *Circles*: Group centroids of the different phylogenetic and phylogeographic clades (according to [33]) included as a supplementary variable. Note that for each of the two genera *Clypeadon* (*orange*) and *Philanthinus* (*red*) only one species was included in the analysis, thus, their object scores are identical to their group centroids. Color code: *yellow*, genus *Cerceris*; *orange*, genus *Clypeadon*; *red*, genus *Philanthinus*; *purple*, Palearctic/Asian *Philanthus*; *blue*, Palearctic/Afrotropical *Philanthus*; *light green*, Nearctic *Philanthus*; *dark green*, genus *Trachypus*. Abbreviations of morphological characters (numbering corresponds to section 2.4.1): ppg.structure, (1) overall structure of the PPG; ppg.size, (2) size of the PPG relative to the head capsule; ppg.parts, (3) modifications of PPG morphology; ppg.branching, (4) branching of the PPG; ppg.open.lower, (5) numbers of openings of the lower part of the PPG to the pharynx; ppg.wall, (6) structure of the inner walls of the PPG; ppg.type, (7) type of gland cells associated with the PPG; mg.presence, (8) presence of the MG; mg.size, (10) size of the MG relative to the head capsule; mg.branching, (12) branching of the MG; mg.type, (14) type of gland cells associated with the MG

Both PPG and MG show remarkable interspecific variation with respect to their incidence, size and shape (Fig. 3), as well as the fine structure of the gland reservoirs and the type and arrangement of the associated secretory cells (Fig. 2). Character states for the species under study are given in Additional file 1: Tables S1 and S2. The CATPCA analysis based on 11 morphological characters (Table S2) sorted the species under study into three well defined groups (I-III, see below) and two species largely separated from these groups (Fig. 4). The first two dimensions of the CATPCA together explained 94% (63% and 31%, respectively) of the variance in the dataset and were supported by a total Cronbach’s α of 0.99 (maximum value = 1), indicating the high reliability of the detected pattern in the dataset [84]. Size and complexity of MG and PPG strongly contribute to the separation of the groups, and their vectors point in opposite directions. Yet, according to phylogenetic independent regression analyses there was no significant correlation between size (*N* = 30, *r* = −0.47, *p* = 0.136) or complexity (*N* = 30, *r* = −0.6, *p* = 0.14) of MGs and PPGs across species. However, due to the comparatively small set of species in our analysis [85] this result bears some uncertainty.

#### Group I: Species possessing large MGs but only small PPGs

The first group of species as assigned by the CATPCA (Fig. 4) is characterized by complex and large MGs, but only small and simple PPGs. In all species of this group, the MG reservoir (turquoise in Fig. 3) opens at the dorsal side of the mandible base and is bordered by a rather thin monolayered epithelium in direct contact with gland cells that show the typical end apparatus of NQ-class 3 cells, but no canals (Fig. 2c). *Cerceris rybyensis* (ID 2) additionally possesses a second reservoir (dark blue in Fig. 3) with a distinctly thicker, yet likewise monolayered epithelium and being exceptional in having two openings, one dorsally and one ventrally at the mandibular base. This additional reservoir is associated with typical NQ-class 3 cells with end apparatus and canals. The small PPG reservoirs of group I species are not associated with any cells that show signs of secretory activity. Notably, group I solely comprises the three investigated species of the tribes Cercerini and Aphilanthopsini (IDs 1-3).

#### Group II: Species possessing both well-developed MGs and PPGs

The second group comprises 12 species (including the two subspecies of *P. triangulum*, IDs 16 and 17) (Fig. 4) that possess both large, complex PPGs and mostly medium-sized, yet well-developed MGs with fairly thick epithelia. Most members of this group possess only the upper part of the MG (dark blue in Fig. 3), whereas *Philanthus* cf. *basalis* (ID 5), *P. t. triangulum* (ID 16), and *P. T. diadema* (ID 17) possess both upper and lower parts and *Trachypus elongatus* (ID 32) possesses only the lower part of the MG (light blue in Fig. 3). In nine species of group II, the MG is associated with acini made up of NQ-class 3 cells with canals jointly connecting an acinus with the reservoir (Fig. 2b). Yet, the closely related *Philanthus histrio* (ID 12) and *P. rugosus* (ID 15) as well as the two *Trachypus* species (IDs 32 and 33) possess single NQ-class 3 cells (Fig. 2a).

In eight species of group II, the PPG reservoir is extensively ramified and associated with cells that show clear signs of secretory activity. In seven of these species the cells at the PPG are syncytia (Fig. 2e); only in *Trachypus flavidus* (ID 33) these cells are aggregations of mononuclear cells. The remaining five species of group II, *P.* cf. *basalis* (ID 5), *P. capensis* (ID 9), *P. t. triangulum* (ID 16), *P. T. diadema* (ID 17), and *T. elongatus* (ID 32) possess large, un-ramified more or less tube-shaped PPGs and neither the cells of the PPG epithelium nor surrounding cells show signs of secretory capacity. Group II comprises all but two of the investigated Palearctic, Indian, and Afrotropical species of the genus *Philanthus* (IDs 5, 7 and 9-17), as well as the two Neotropical species *T. elongatus* (ID 32) and *T. flavidus* (ID 33).

*Trachypus patagonensis* (ID 34) that was not included in the CATPCA (see "Data matrix for statistical analysis") would probably also be placed in this group. Its MG consists of both upper and lower part associated with single NQ-class 3 cells and its PPG is tubular and not associated with secretory cells.

#### Group III: Species with large, complex PPGs but no MGs

The third group is rather narrowly defined and comprises 14 *Philanthus* species characterized by completely lacking an MG but possessing large and extensively ramified PPGs (Fig. 3) associated with secretory cells. *Philanthus venustus* (ID 8) deviates from the other members of group III in that the secretory cells of its PPG are not syncytia but aggregations of mononuclear cells (Fig. 2 d), similar to *T. flavidus* (ID 33) in group II. Only in species of group III are the syncytia or cell aggregations associated with the PPG branches interspersed with small rounded cells with barely any cytoplasm (white arrows in Fig. 2d and f). Most species of group III have a Nearctic distribution, the exceptions being the Indian *Philanthus pulcherrimus* (ID 6) and the Palearctic *Philanthus venustus* (ID 8).

#### Divergent species

Two species included in the CATPCA are separated from the three main groups. One is *P. multimaculatus* (ID 24), the only Nearctic species in our dataset whose males have an MG. Like the Neotropical *T. elongatus* (ID 32) it has only the lower part of the MG (Fig. 3). In the CATPCA it is located between its MG-less Nearctic relatives of group III and the Afrotropical, Palearctic and Neotropical species of group II that all possess MGs. The second separated species is *P. quattuordecimpunctatus* (ID 4), whose males have a well-developed tube-shaped MG, associated with cells akin to NQ-class 3 gland cells that, however, lack conducting canals, resembling group I in this respect. The upper part of their PPG extends backwards around the brain, like in the species of group II, and is not associated with any secretory cells. Moreover, the PPG of *P. quattuordecimpunctatus* is unique among all investigated species in that its reservoir consists of an complex network of lamellae (not shown) as opposed to the tubular ramifications of the other species.

*Philanthus albopilosus* (ID 18; not in CATPCA, see "Categorical principal components analysis" and Discussion) stands out from all other species. Its males not only completely lack an MG, like most of their Nearctic congeners, but also have a largely reduced PPG that consists of only small evaginations of the pharynx (Fig. 3) without any secretory cells, similar to the PPGs of group I.

### Phylogenetic trend in gland morphology

**Fig. 5:**
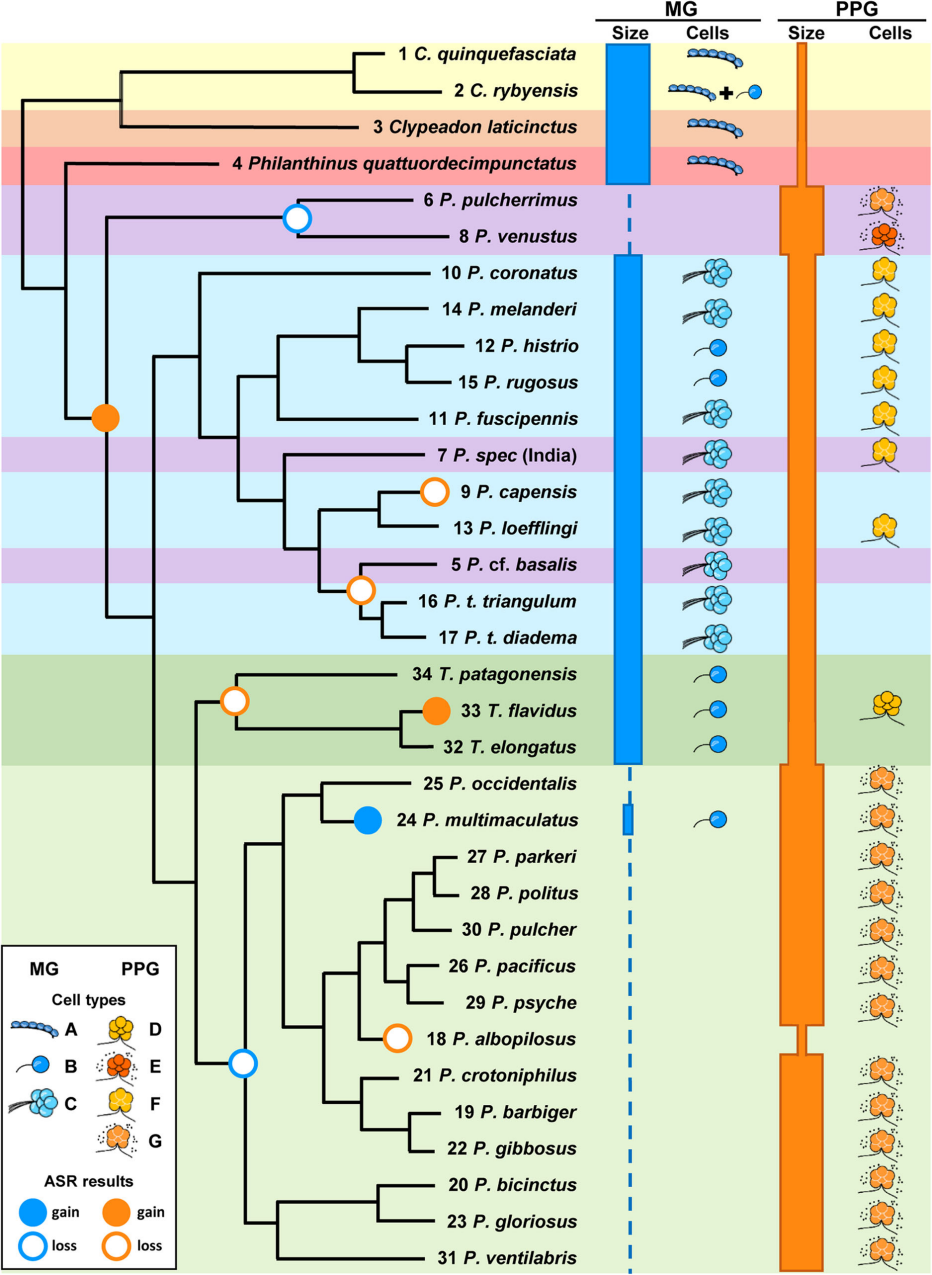
Summary of the phylogenetic trends and deviations in size of the MG and PPG (*bar thickness* indicates relative size, dotted line indicates absence) and type of associated gland cells (‘Cells’) among male Philanthinae. *Circles at nodes* indicate likely events of gain or loss of the MG or the PPG (symbols see key in figure; for more details see Additional file 1: Figure S4 and S5). Species IDs correspond to Table 1. Color code of phylogeographic clades (according to [33]): *yellow*, genus *Cerceris*; *orange*, genus *Clypeadon*; *red*, genus *Philanthinus*; *purple*, Palearctic/Asian *Philanthus*; *blue*, Palearctic/Afrotropical *Philanthus*; *light green*, Nearctic *Philanthus*; *dark green*, genus *Trachypus*). Pictograms of cell types (labeling see key in figure): (*A*) single gland cells, showing end apparatuses but directly associated with the wall of the MG reservoir without canal cells (*B*) single NQ-class 3 gland cells; (*C*) acini of several NQ-class 3 cells with bundles of conducting canals; (*D*) aggregations of several gland cells directly associated with very fine branches of the PPG; (*E*) as in (D), but interspersed with small rounded cells; (*F*) syncytia of secretory cells directly associated with very fine branches of the PPG; (*G*) syncytia as in (*E*), but interspersed with small rounded cells. Dendrogram modified after the molecular phylogeny of Kaltenpoth et al. [33]

As summarized in Fig. 5, the gland morphology of male Philanthinae partly coincided with phylogenetic groups, but there is also considerable diversity within clades and several species deviate from their closest relatives. To test whether there is an overall phylogenetic trend in gland morphology we conducted a HCA (Additional file 1: Figure S3) based on the morphological characters of MG and PPG and compared the resulting dendrogram with the molecular phylogeny of the Philanthinae [33]. The HCA largely corroborated the pattern found in the CATPCA (for details on the clustering of species see Additional file 1). Notably, the HCA dendrogram shows a highly significant congruency with the molecular phylogeny (cophylogenetic analysis, all tested parameter combinations: *p* < 0.001).

### Phylogenetic history of the MG

The MG shows a complex phylogenetic pattern of incidence among male Philanthinae (Fig. 5). A maximum likelihood ASR using an unbiased model (bias = 1, Additional file 1: Figure S4) suggests the presence of an MG as the ancestral state of the subfamily Philanthinae as well as of the tribe Philanthini and of the genus *Philanthus* (including *Trachypus*). Accordingly, the MG would have been independently lost twice within the genus *Philanthus*, namely in the last common ancestor of the clade comprising *P. pulcherrimus* (ID 6) and *P. venustus* (ID 8) (ML probability 96%) and in the last common ancestor of the Nearctic *Philanthus* species (ML probability 100%) (Additional file 1: Figure S4). In the Nearctic *P. multimaculatus* (ID 24), however, the MG must have been regained (Additional file 1: Figure S4). This result did not change if losses were assumed to be more frequent than gains (bias <1) and also if gains were assumed to be slightly more likely than losses (up to a bias of 1.5). Varying the bias further in favor of gains (bias ≥2.3), however, led to ambiguous or deviating results for some nodes within the genus *Philanthus* (including *Trachypus*) (Additional file 1: Figure S4). Yet, even with a bias of 10 the analysis indicated the presence of an MG as the ancestral state for both the Philanthinae and the Philanthini (Additional file 1: Figure S4).

### Phylogenetic history of the secretory cells of the PPG

The phylogenetic pattern of the presumed secretory cells of the PPG is even more complex (Fig. 5). According to an unbiased maximum likelihood ASR (bias = 1), the secretory cells of the PPG were most likely absent in the last common ancestors of both the Philanthinae and the Philanthini and first occurred in the last common ancestor of *Philanthus* (including *Trachypus*) (ML probability 100%; Fig. 5, Additional file 1: Figure S5). Within *Philanthus*/*Trachypus*, the secretory cells would then have been independently lost four times (Fig. 5, Additional file 1: Figure S5), namely in the last common ancestor of the Neotropical *Trachypus* (IDs 32-34) (ML probability 71%), in the Nearctic *P. albopilosus* (ID 18), in the last common ancestor of the clade containing the two subspecies of *P. triangulum* (IDs 16 and 17) and *P.* cf. *basalis* (ID 5) (ML probability 87%) as well as in the related Paleotropical *P. capensis* (ID 9), while the closely related *Philanthus loefflingi* (ID 13) has retained the secretory cells. Hence, one species, *T. flavidus* (ID 33), must have regained the secretory cells of the PPG (Additional file 1: Figure S5). Varying the bias strongly in favor of gains over losses (bias ≥4), resulted in a somewhat different evolutionary scenario in that the secretory cells of the PPG would have been lost in the last common ancestor of both the *P. capensis*-clade and the *P. triangulum*-clade and then regained in *P. loefflingi* (Additional file 1: Figure S5).

### Comparison of morphological diversity in males and females

The CATPCA based on the aggregate matrix of character states for males and females reveals a clear distinction between the sexes (Additional file 2: Figure S6). Whereas data points for females are largely clumped, the data points for males are much more scattered and show two main aggregations similar to the CATPCA including only males. Diversity estimates of character states among characters of gland morphology were significantly higher in males (mean ± s.d.: 1.12 ± 0.27) than in females (0.34 ± 0.32; Wilcoxon matched pairs test: *N* = 9 characters, exact *p* = 0.004).

## Discussion

There are several comparative phylogenetic studies on secondary sexual traits (e.g. [28, 29, 86–90]), but the present study is, to our knowledge, the first comparative histological study on insect exocrine glands that are under sexual selection. Males of all but one of the investigated Philanthinae bear enormous and elaborate head glands that are considerably larger and more complex than in females of any species of this subfamily [59]. The exaggeration of the male glands emphasizes their significance for mate attraction and the strength of sexual selection acting upon them.

Our comprehensive investigation revealed considerable interspecific variation with numerous species deviating from their close relatives with regard to gland occurrence, size and morphology, as well as the incidence, specific type and arrangement of associated secretory cells. Nevertheless there was a clear phylogenetic trend in gland morphology (summarized in Fig. 5). Ensuing from a plesiomorphic state, two main evolutionary trends emerge: First, the PPG increases in size and complexity and becomes involved in the production and storage of the marking secretion. Second, the MG, in return, decreases in size and is eventually completely lost.

### The plesiomorphic state of the Philanthinae

About half of the species under study lacked an MG. To shed light on the plesiomorphic state of the subfamily, we conducted an ancestral state reconstruction. The most likely scenario is the presence of an MG in the predecessor of the Philanthinae, of the Philanthini and of the genus *Philanthus* (including *Trachypus*) with repeated losses in single lineages and one gain (Fig. 5, Additional file 1: Figure S4). This view is corroborated by the fact that such ectal MGs [91–93] as found in male Philanthinae occur in all major lineages of the Aculeata (bees: e.g. [94–96], apoid wasps: [93, 97], vespid wasps: [92], ants: e.g. [98, 99]) and in parasitoid wasps [100, 101]. Moreover, females of all investigated Philanthinae have MGs, albeit small [59], indicating that the genetic information to develop this gland is present throughout the subfamily. Males of all studied species possess a PPG that is probably homologous to the PPGs of ants (Formicidae) [52, 102] and the cockroach wasp *Ampulex compressa* (Ampulicidae) [103]. In the majority of species under study, the PPG is associated with secretory cells. An ASR for the occurrence of these cells revealed that they were probably absent in the last common ancestor of Philanthinae and Philanthini. Accordingly, these cells must have evolved in the last common ancestor of *Philanthus*/*Trachypus*, but were lost several times within this taxon and regained at least once (Fig. 5, Additional file 1: Figure S5).

The inferred plesiomorphic state of male Philanthinae is represented by the investigated Cercerini and Aphilanthopsini with their large MGs and small PPG reservoirs devoid of secretory cells (Fig. 5). The MGs of these species share a type of gland cells that bear end apparatus but, in contrast to typical NQ-class 3 cells, do not show canals. Such gland cells seem uncommon, but have been described for an ant [104] and some bee species [97]. Notably, there is some variation among the Cercerini in that *C. rybyensis* males have an additional part of the MG with typical NQ-class 3 cells. Our results on *C. rybyensis* are largely consistent with Ågren [56], who, however, did not mention the gland cells with end apparatus but no canals. The enormous size of the MG reservoir of male Cercerini and Aphilanthopsini and the high number of associated secretory cells suggest that the function of the MG comprises both production and storage of the male marking secretion. In other taxa of Hymenoptera, the MG is known as source of different pheromones like male and female sex pheromones [101, 105–108], the queen pheromone in honeybees (*A. mellifera*) (e.g. [109, 110]) and alarm pheromones in different ants (e.g. [111–113]). The MG can also be the source of defensive secretions in parasitoid wasps [101], bees [114, 115], and ants [116].

The PPGs found in male Cercerini and Aphilanthopsini in the present study largely resemble the PPGs of the respective conspecific females [59]. Moreover, the shape and structure is quite similar to the PPGs of both sexes of the cockroach wasp *A. compressa* [103], a rather basal taxon within the Apoidea [117, 118]. Notably, a PPG had not previously been described for male Cercerini and Aphilanthopsini and currently no information is available on their chemistry. Considering their small size and the lack of secretory cells, we hypothesize that in these tribes the males’ PPGs do not play an important role in the production and/or storage of a marking secretion. Instead, as suggested for *A. compressa* [103], the PPG may function as a hydrocarbon reservoir. Until recently, a PPG was only known from ants where it mainly serves to generate the colony odor that is also based on hydrocarbons [119–122] (for a review of other functions of the PPG in ants, see [123]). Such a “social function” of the PPG can be ruled out for the solitary Cercerini and Aphilanthopsini.

### The involvement of the PPG

The head glands of male Philanthini differ markedly from the Cercerini and Aphilanthopsini since their MGs are more or less reduced and their PPGs are typically considerably larger and more complex (Fig. 5). Like in most Hymenoptera (e.g. [74, 100, 101, 124–126]), the MGs of male Philanthini are exclusively associated with typical NQ-class 3 cells, either in single units or arranged in acini. As in *P. triangulum* [50–52], the male MG of other Philanthini is presumably also involved in the production of the marking pheromone.

Taking into account its position at the very base of the Philanthini, the genus *Philanthinus* may be expected to represent an intermediate state between the Cercerini and Aphilanthopsini and the Philanthini. In fact, the somewhat smaller MG with typical NQ-class 3 gland cells and the large PPG of *P. quattuordecimpunctatus* (Fig. 3) support this view. However, in contrast to most *Philanthus* species its PPG is not associated with secretory cells (Fig. 5) and *P. quattuordecimpunctatus* stands out from all other Philanthinae with regard to the structural organization of the PPG in lamella-like branches.

In the genus *Philanthus*, males of nearly all studied species possess at least moderately large PPGs (Fig. 5, see also Fig. 3). In most species these PPGs shows extensive ramifications that are closely associated with cells (syncytia or, rarely, cell aggregations) that show clear signs of secretory activity, like large nuclei with several nucleoli and numerous vesicles. Even though these cells do not conform to any previously described type of secretory cell [71, 127, 128], we hypothesize that they synthesize compounds of the marking secretion that are transferred to the PPG reservoir, where they are stored until release during territory marking. How these cells evolved and whether their secretion is transported to the PPG lumen by direct contact as suggested by their close proximity to the PPG ramifications is not known yet. Notably, in species that have lost these secretory cells associated with the PPG (*P. t. triangulum*, *P. t. diadema, P.* cf. *basalis*, *P. capensis*, *T. elongatus*, and *T. patagonensis*) the PPG reservoirs consist of voluminous tubes without ramifications that presumably merely store the marking secretion that is produced in the MG [50–52]. Inspection of the mapping of PPG characters on the phylogeny suggests that the secretory cells and the elaboration of the PPG reservoir may have evolved concurrently at the base of the genus *Philanthus* (Fig. 5).

Our results suggest that the PPG contributes to a variable degree to pheromone storage and production in males of most Philanthini. So the question arises why and how its involvement in scent marking came about. Beewolf females have been observed to simply alight in a male’s territory and allow mating without additional courtship by males [37, 47]. Therefore, the conspicuousness of the territory, mediated by the composition and amount of marking pheromone, is probably the most important determinant for male reproductive success. Moreover, the spatial proximity of scent marking males in leks, as has been shown for several *Philanthus* species [37, 46, 47], might allow females to directly compare territories and their owners. This results in strong sexual selection on males to maximize both the quantity and quality of the marking secretion.

The original dual role of the MG as site of synthesis and reservoir of the marking secretion (as found in the Cercerini and Aphilanthopsini) might have limited the ability of males to synthesize and store larger amounts of marking secretion or to add novel compounds to the blend. For example, novel classes of compounds might have interfered with the synthesis or storage of the existing components (e.g. due to chemical reactions between acids and alcohols), thus promoting the evolution of novel secretory cells and a separate reservoir. The first evolutionary step towards its prominent role in scent marking might thus have been a minor participation of the PPG in the storage and production of the marking secretion. Ongoing selection on pheromone quantity and quality would subsequently have enlarged the PPG and augmented its contribution. Whether the involvement of the PPG to pheromone production is accompanied by changes in the chemical composition of marking secretions in the Philanthini, in particular by the addition of novel classes of compounds, should be revealed by a comparative study of the marking secretions among the Philanthini.

An increase in the amount of scent marking secretion would clearly have been an advantage for mate attraction [129–131]. The addition of novel components to a sex pheromone, however, may represent a saltational evolutionary change [30], potentially even hindering mate recognition. Novel compounds might nevertheless be selected for by several not mutually exclusive causes like predation avoidance, male-male competition, and female choice [30, 132, 133]. There is currently no evidence that male scent marking in the Philanthini is effective in repelling predators or in keeping conspecific males at bay. However, different processes related to female choice might explain the evolution of novel pheromone components. First, female sensory biases [134–137] that evolved for prey recognition purposes might influence pheromone composition as in *P. triangulum* [53–55]. Consequently, a shift in the females’ prey spectrum might select for changes in the males’ marking secretion. Second, Fisher-Zahavi processes [23–25] could cause the addition of novel components. In Fisher’s run-away model a female preference might arise accidentally and coevolve with the preferred trait; but this process has rarely been considered for pheromone evolution. Female choice for good or compatible genes could affect the evolution of pheromones [31], in that new components could indicate additional aspects of male quality [138] or improve signal reliability [139]. Finally, since sympatry is widespread among *Philanthus* species (e.g. [37]; G. Herzner, E. Strohm, M. Kaltenpoth, unpublished) the establishment or reinforcement of reproductive isolation between species [30, 140–142] might have selected for novel pheromone components [31].

If the involvement of the PPG enhanced mate attraction in male Philanthini, the question arises why the PPG did not get involved in scent marking (and was not enlarged) in the Cercerini and Aphilanthopsini as well. One possible explanation is that males of these tribes experience weaker sexual selection because, compared to male Philanthini, they have less pronounced territorial behavior and are spatially more dispersed [37, 38, 41, 42, 143]. Different intensities of sexual selection on males could also explain that PPG morphology shows a conspicuously congruent pattern in both sexes among the Philanthinae, with smaller PPGs in the Cercerini and Aphilanthopsini and larger, more complex PPGs in the Philanthini [59]. Owing to correlated evolution between the sexes [144–147], genetic changes underlying the sexually selected elaboration of the PPG in male Philanthini, as documented in this study, could have facilitated an enlargement of the PPG and the evolution of prey embalming in female Philanthini [58, 59, 61–63, 65]. That the PPG has evolved independently in males and females and the observed congruency across tribes is merely accidental seems rather unlikely. Yet, another plausible scenario is that the initial augmentation of the PPG might first have evolved in female Philanthini due to strong natural selection for prey embalming [58, 59, 61–63, 65] and, again assuming correlated evolution between the sexes [144–147], the resulting genetic changes could have facilitated the subsequent enlargement and elaboration of the PPG in male Philanthini by sexual selection. Different natural selection pressures on female Cercerini and Aphilanthopsini [59] may have kept the PPGs of both sexes of these basal tribes comparably small and simple.

### The loss of the MG

The loss of the MG in the Nearctic *Philanthus* was surprising, since MGs had previously been reported from males of five of these species [48, 148–150]. We suspect that in these studies the large PPGs were mistaken for MGs, because their conclusions were based on dissections that hardly allow the discrimination of the two glands and at that time PPGs were only known from ants [50, 52].

Notably, in all but one species without MG the PPGs are huge and show extensive ramifications in direct contact with multinuclear syncytia (or aggregations of cells in *P. venustus*) (Fig. 5). Only in species lacking the MG (and in *P. multimaculatus*) the PPG is interspersed with conspicuous small cells (Fig. 5, see also Fig. 2d and f). This might suggest that these cells substitute for some function of the MG. However their small size and little cytoplasm contradict a secretory capacity. Due to the size and complexity of the PPGs and their association with large secretory cell clusters, we conclude that in the species without MGs, the PPG alone is responsible for the synthesis and storage of the marking secretion.

While it appears plausible that the enlargement of the PPG caused a reduction of the MG, its complete loss in several clades of the Philanthini is most puzzling, because it might have been accompanied by the loss of certain components of the marking secretion. Non-adaptive explanations like genetic drift in small populations could hardly explain the disappearance of a whole gland system. According to the above mentioned idea that the involvement of different glands is driven by hybridization avoidance, the loss of components of a sex pheromone and the respective gland might be possible if the risk of hybridization is lowered. However, since particularly Nearctic species often occur in sympatry (e.g. [37]; G. Herzner, E. Strohm, M. Kaltenpoth, unpublished), a reduced risk of hybridization compared to other clades seems unlikely. An alternative explanation is that a change in female preferences to compounds that can be more efficiently produced in the PPG might make an MG superfluous. Female preferences [134–137] might be altered because of a change in their prey spectrum as explained above. In many Nearctic *Philanthus*, females prey not only on bees but also on wasps, whereas the latter habit seems to be rare in Palearctic and Afrotropical species [37]. Whether such a difference could cause the loss of the MG in males of the Nearctic species cannot be answered yet. Otherwise, there are no conspicuous differences between the Nearctic species and their Palearctic/Afrotropical congeners with regard to scent marking and reproductive behavior [37] that could explain the loss of the MG. Unfortunately, very little is known about the other two species without MG, *P. venustus* and *P. pulcherrimus*.

The loss of a sexual character is becoming increasingly recognized as a common event in the evolution of sexually selected traits and may have different causes [135, 151]. In beewolves, however, the actual trait, scent marking, persists while the source of the secretion is changed. A similar phenomenon has been reported for solitary bees of the genus *Centris*. Depending on the species, males scent mark territories with a secretion from either the MG or tibial glands and the respective other gland is reduced [105, 106, 152].

### Taxa deviating from the overall trend

*Philanthus albopilosus* is the only known species of the genus in which males do not establish and scent mark territories [37]. Therefore, they do not need the respective glands anymore and their PPG has been reduced (Fig. 5). This provides indirect evidence for the role of the PPG in the production and storage of the marking secretion in other male Philanthini. The reduction of a gland following the loss of its function has been reported for fungus-growing ants. In monandrous attine ants, males transfer an antiaphrodisiac from accessory glands during copulation; in polyandrous species, however, males do not mark mated queens and their accessory glands were reduced or completely lost [153].

The regain of the MG in males of the Nearctic *P. multimaculatus* (Fig. 5) is puzzling since there are no conspicuous differences to its Nearctic congeners with regard to their territorial behavior [37]. Also, why in some species (*P. triangulum, P.* cf. *basalis, P. capensis*) the secretory cells of the PPG were lost while the reservoir became the main storage organ (Fig. 5) cannot be answered yet.

## Conclusion

There is substantial evidence that sexually selected traits can undergo rapid evolutionary change, including losses and gains [83, 154–156]. In particular the Fisher-Zahavi processes [23–25] as well as sexual antagonism, like chase-away selection [26] and female sensory biases [135–138] might cause complex phylogenetic patterns in sexually selected characters (e.g. [28, 87–89, 157–159]). Our comparative morphological analyses of male head glands revealed extensive interspecific variation within the Philanthinae, in particular among the Philanthini. While we found clear phylogenetic trends, there are also intriguing deviations and reversals (Fig. 5). The glands of female Philanthini, by contrast, appear virtually uniform with mostly only gradual variation and no loss of a gland system or the addition of novel components like secretory cells [59], probably as a result of stabilizing natural selection. Other evolutionary forces like genetic drift and mutations should affect males and females similarly and can therefore be excluded as causes for the observed higher diversity among males. Taken together our findings support the hypothesis that strong sexual selection acting on male pheromone glands has led to rapid evolutionary changes and to a substantially higher interspecific morphological diversity in males than in females. Taking into account that about 135 of the ca. 170 described species of Philanthini [68] have not been investigated so far, the high diversity observed in this study suggests that there are probably more species with unique and novel gland characteristics yet to be discovered. Further studies on the chemical composition of the marking secretions, male territorial behavior, mate attraction as well as female prey spectrum and mate choice will help to unravel the ecological and evolutionary causes that have given rise to the remarkable diversity and phylogenetic trends in male head gland morphology among the Philanthinae.

## Additional files

Additional file 1: Additional methods, additional **Table S1.** showing the data matrix of the analyzed morphological characters, additional **Table S2.** showing the data matrix used for statistical analyses, additional **Table S3.** giving the Eigenvalues of the morphological characters from the categorical principal components analysis, additional **Figure S1.** explaining the morphology of the PPG, additional **Figure S2.** showing sagittal section of the head capsule of *Philanthus rugosus*, additional results of the hierarchical cluster analysis based on PPG and MG morphology, including additional **Figure S3.** showing the dendrogram resulting from the hierarchical cluster analysis, additional **Figure S4.** showing the results of the ancestral state reconstruction of presence vs. absence of the MG, and additional **Figure S5** showing the results of the ancestral state reconstruction of presence vs. absence of secretory cells of the PPG. (PDF 1617 kb)

Additional file 2: Additional methods for coding of morphological characters for the aggregated analysis of male and female head gland morphology, additional **Table S4.** showing the aggregated data matrix used for statistical analyses of male and female gland morphology, additional information on the aggregated categorical principal components analysis, including additional **Table S5.** giving the Eigenvalues of the aggregated morphological characters from the categorical principal components analysis of male and female gland morphology, additional **Figure S6.** showing the plot of the aggregated categorical principal components analysis, and additional methods on the calculation of Shannon diversity indices for the aggregated characters. (PDF 739 kb)

## Abbreviations

3D: Three-dimensional
ASR: Ancestral state reconstruction
CATPCA: Categorical principal components analysis
HCA: Hierarchical cluster analysis
ID: Identification number of species
MG: Mandibular gland
ML: Maximum likelihood
NQ-class: Class of secretory cell according to Noirot and Quennedey 1974 (71)
PPG: Post pharyngeal gland
