# Supplementary material for: Sexual selection and the evolution of male pheromone glands in philanthine wasps (Hymenoptera, Crabronidae)
Source: BMC Evol Biol. 2017 Jun 6;17:128. doi: 10.1186/s12862-017-0963-6 (PMC5461632; doi:10.1186/s12862-017-0963-6)
Supplement: Supplementary file 1 — Additional methods, additional Table S1. showing the data matrix of the analyzed morphological characters, additional Table S2. showing the data matrix used for statistical analyses, additional Table S3. giving the Eigenvalues of the morphological characters from the categorical principal components analysis, additional Figure S1. explaining the morphology of the PPG, additional Figure S2. showing sagittal section of the head capsule of Philanthus rugosus, additional results of the hierarchical cluster analysis based on PPG and MG morphology, including additional Figure S3. showing the dendrogram resulting from the hierarchical cluster analysis, additional Figure S4. showing the results of the ancestral state reconstruction of presence vs. absence of the MG, and additional Figure S5 showing the results of the ancestral state reconstruction of presence vs. absence of secretory cells of the PPG. (PDF 1617 kb) [file 12862_2017_963_MOESM1_ESM.pdf]

Additional File 1

Sexual selection and the evolution of male pheromone glands in philanthine wasps (Hymenoptera, Crabronidae)

Katharina Weiss, Gudrun Herzner, Erhard Strohm*

Evolutionary Ecology Group, Institute of Zoology, University of Regensburg, Universitätsstr. 31, 93053 Regensburg, Germany

* Corresponding author: Erhard Strohm
Universitätsstr. 31
93053 Regensburg
Germany
Phone: +49-941-943-3072
Fax: +49-941-943-3304
Erhard.Strohm@ur.de

Email addresses: Katharina1.Weiss@ur.de (Katharina Weiss)
Gudrun.Herzner@ur.de (Gudrun Herzner)
Erhard.Strohm@ur.de (Erhard Strohm)

Additional Methods

1 Definition of morphological characters and coding of character states and phylogenetic/ phylogeographic affiliation

Based on the comprehensive examination of both semithin histological sections and 3D-reconstructions of the head glands of males of 33 species and one subspecies of the Philanthinae (Table 1, main text), we defined 14 morphological characters for the PPG and MG, as well as associated secretory cells. For each character, different states were categorized and numerically coded for the statistical analysis as described below. Note that for characters 2, 4, 5, 10, and 12 the character states can be ranked and thus represent ordinal data. Moreover, for all statistical analyses for characters 3, 4, 9, and 13 some categories were pooled as specified below ('combined dataset', Table S2; see also main text: "Data matrix for statistical analysis").

PPG:

1 *Overall structure of the PPG reservoir*.

- **(0)** PPG consists of both an upper reservoir originating from the dorsal side of the pharynx and a lower evagination originating ventrally from the pharynx
- **(1)** PPG consists of only the upper reservoir

2 *Relative size of the upper PPG reservoir*. In male Philanthinae, the paired reservoirs of the upper PPG originate dorsally from the pharynx and extend backwards subjacent to the cuticle of the head capsule. In species with large PPGs, the reservoirs reach around the brain and may even proceed to the front of the head capsule ventrally to the brain, in some cases almost reaching the mandible base. The size of the upper PPG reservoir is specified as its longitudinal extension in relation to the size of the head capsule. This estimate is only slightly altered by the filling status of the gland, since this mainly affects the lateral extension of the gland. After a preliminary analysis we defined three size classes. The limits of these size classes follow a geometric progression with the ranges of the size classes increasing by the factor 2. This allowed for a simple and reliable classification and covered the spectrum of cases.

- **(0)** small PPG (spanning approximately 25 - 50% of the head capsule)
- **(1)** medium sized PPG (spanning approximately 50 - 100% of the head capsule)
- **(2)** large PPG (spanning approximately 100 - 200% of the head capsule, i.e. it reaches around the brain and extends downwards towards the mandible base ventrally to the brain)

3 *Modifications of the PPG morphology*. In all investigated male Philanthinae the main part of the PPG comprises paired lateral reservoirs of the upper gland part extending dorsally above and, depending on its size, around the brain (dPPG in Fig. 1, main text). In most species these reservoirs

are accompanied by a second (often smaller) set of paired reservoirs branching off from the dorsal reservoirs and extending somewhat lateral and anterior to the brain (aPPG in Fig. 1, main text). Some species possess an additional considerably smaller unpaired part of the PPG consisting of a ventral evagination of the pharynx (see character 1). However, most species show modifications of this basic pattern that could be clearly differentiated. We distinguished six such morphological modifications of the PPG, which were denoted A - F and are described in Fig. S1. The species under study exhibited different combinations of these modifications. For the 'combined dataset', categories (7) and (8) were pooled (Table S2).

(0) A

(1) A + E

(2) A + F

(3) A + B

(4) A + B + E + F

(5) A + B + C

(6) A + B + C + F

(7) A + B + C + E + F

(8) B + D + E + F

4 *Branching of the PPG reservoir*. The main reservoir of the upper PPG can be branched to a variable extent, ranging from unbranched reservoirs, not associated with gland cells, to extensively branched reservoirs with numerous fine branches, which are directly associated with aggregations of mononuclear cells or syncytia of secretory cells (see character 7). For the 'combined dataset', categories (2) - (4) were pooled (Table S2).

(0) unbranched

(1) few voluminous branches (without fine branches)

(2) some fine branches

(3) many fine branches

(4) very many fine branches

5 *Number of openings of the lower part of the PPG to the pharynx*.

(0) one opening

(1) two openings

6 *Structure of the inner walls of the PPG reservoir*.

(0) unstructured

(1) cuticular ripples

(2) hairs

7 *Type of gland cells associated with the PPG*. In 23 of the investigated 33 species of Philanthinae the PPG reservoir is associated with cells with a presumably secretory function. However, these cells do

not resemble any of the gland cells classes as defined by Noirot and Quennedey [1]. These cells could be assigned to clearly definable categories.

(0) no gland cells

(1) PPG-type 1: several gland cells are tightly associated and form cell aggregations that are directly associated with very fine branches of the PPG (see character 4)

(2) PPG-type 2: similar to PPG-type 1, but the cell aggregations are interspersed with small rounded cells (Fig. 2 D, main text)

(3) PPG-type 3: the secretory cells consist of syncytia (i.e. aggregations of cells but no cell membranes visible between cells) that are directly associated with very fine branches of the PPG (see character 4) (Fig. 2 E, main text)

(4) PPG-type 4: similar to PPG-type 3 but the syncytia are interspersed with small rounded cells (similar to those described for PPG-type 2) (Fig. 2 F, main text)

In the preliminary investigation, we also recorded the number of openings of the upper part of the PPG to the pharynx. However, this character was invariant with all investigated species possessing a separate opening for each of the two sides of the upper PPG and was therefore not included in the dataset.

MG:

8 *Presence of the MG reservoir*.

(0) no MG reservoir

(1) MG reservoir present

9 S*tructure of the MG reservoir*. In species of the tribe Philanthini, the MG can consist of an upper and a lower reservoir, which open dorsally and ventrally to the mandible, respectively. In male Cercerini and Aphilanthopsini, the MG likewise originates dorsally to the mandible, but possesses a thinner and less structured wall as compared to the MG of the Philanthini. In one species, *Cerceris rybyensis*, this thin-walled MG reservoir occurs alongside a small reservoir resembling the upper MG of the Philanthini. We regarded the thin-walled upper MG of the Cercerini and Aphilanthopsini as a distinct part of the MG (categories (0) and (1) in the 'full dataset', Table S1). For the 'combined dataset', however, categories (0) and (1) were pooled with category (2) (Table S2).

(0) only the thin-walled MG reservoir is present

(1) in addition to the thin-walled MG (0), a second MG reservoir with thicker walls (like in (2)-(4)) is present

(2) only the upper part of the MG reservoir is present

(3) only the lower part of the MG reservoir is present

(4) both upper and lower parts of the MG is reservoir present

10 *Relative size of the MG reservoir*. The MG extends from the mandible base laterally and dorsally throughout the head capsule, either proximal or lateral to the PPG reservoir. In some cases, the MG reaches around the brain and can even extend to the front part of the head capsule ventrally to the brain. Hence, analogous to the PPG, we determined the size of the MG relative to the head capsule.

(0) small MG (spanning approximately 25 - 50% of the head capsule)

(1) medium sized MG (spanning approximately 50 - 100% of the head capsule)

(2) large MG (spanning approximately 100 – 200% of the head capsule, i.e. it reaches around the brain and extends downwards towards the mandible base ventrally to the brain)

11 *Location of the MG reservoir in the head capsule*. Depending on its size (see character 10) and on which part of the gland is developed (see character 9), parts of the MG can either be located only in the front part of the head capsule anterior to the brain (A), reach further into the head capsule dorsally to the brain (D), cover the full extension of the head capsule ending behind the brain (B), and/or extend ventral around the brain (V).

(0) A

(1) A + D

(2) A + D + B

(3) A + D + B + V

12 *Branching of the MG reservoir*.

(0) unbranched: the MG consists of paired sac-like evaginations as reservoirs

(1) low: the MG consists of paired reservoirs, each with a few (2-5) distinct branches

(2) high: the MG consists of paired reservoirs, each with many (>5) branches

13 *Structure of the inner walls of the MG reservoir*. For the 'combined dataset', categories (2) - (4) were pooled (Table S2).

(0) unstructured

(1) bearing scattered thin hairs

(2) bearing many thick hairs

(3) bearing several hairs that jointly originate from the tops of flat cuticular sockets, giving the impression of small bushes in the sagittal semithin sections

(4) bearing several ramified hairs that originate laterally from pointed cuticular spines, giving the impression of small trees in the sagittal semithin sections

14 *Type of gland cells associated with the MG*. In all of the 19 investigated species possessing an MG, the reservoir is associated with gland cells. These cells can be clearly assigned to one of several categories. As stated in the main text, section "Histology", we will refer to gland cells corresponding to one of the classes defined by Noirot and Quennedey [1] as 'NQ-class' cells, but we found additional gland cell types that did not match these definitions.

(0) MG-type 1: single gland cells, showing end apparatus but no canals (Fig. 2 C, main text)

(1) MG-type 2: single typical NQ-class 3 gland cells with end apparatus and canal (Fig. 2 A, main text)

(2) MG-type 3: same as MG-type 2, but arranged in acini, i.e. several NQ-class 3 cells are tightly arranged in cell clusters and jointly connected to the MG reservoir via a bundle of canals (Fig. 2 B, main text)

(3) MG-type 4: MG-type 1 and MG-type 2 cells associated with different parts of the MG

Supplementary variable for categorical principle components analysis (CATPCA):

Phylogenetic/phylogeographic affiliation. The subfamily Philanthinae consist of eight genera separated into the three tribes Cercerini, Aphilanthopsini, and Philanthini [2]. According to a recent molecular phylogeny [3], the tribe Philanthini can be divided into several monophyletic clades, largely reflecting their geographic distribution: The genus *Philanthinus* forms a basal sister clade to the Palearctic, Afrotropical, and Indian species of the genus *Philanthus*, from which the Nearctic *Philanthus* species, as well as the Neotropical genus *Trachypus* branch off (thus, *Trachypus* constituting a monophyletic sister group of the Nearctic *Philanthus*, rather than a separate genus [3]). The general phylogenetic affiliation of each species and the geographic origin of species of the Philanthini were coded accordingly (see also dendrograms in Fig. S3 and S4 below) and included into the CATPCA as a supplementary variable (see section 2.1 below).

(0) tribe Cercerini: genus *Cerceris*

(1) tribe Aphilanthopsini: genus *Clypeadon*

(2) tribe Philanthini: genus *Philanthinus*

(3) tribe Philanthini: basal Palearctic/Indian *Philanthus* clade

(4) tribe Philanthini: main Palearctic/Afrotropical *Philanthus* clade

(5) tribe Philanthini: Nearctic *Philanthus* species

(6) tribe Philanthini: genus *Trachypus*

2 Statistical analysis of gland morphology

2.1 Categorical principal components analysis

Analogously to a linear principal components analysis, CATPCA is a data mining tool to search for patterns of variation in a large set of (more or less correlated) variables by reducing them to a small number of uncorrelated principal components, thus facilitating their interpretation while retaining as much as possible of the information in the original data [4, 5]. Yet a CATPCA has two important advantages over its linear equivalent. As the initial analysis step of the CATPCA is the so called nonlinear optimal scaling transformation of the subjected variables, this method can be used to analyze ordinal and nominal variables by replacing the discrete categories of these variables with numeric values called category quantifications. Moreover, depending on the specified analysis level of the transformation, it can also detect nonlinear relationships between variables [4-6].

The 'full dataset' (Table S1) contains all character states as they were recorded. In the 'combined dataset' (Table S2) that was used for statistical analyses, we pooled character states wherever reasonable. Actually, the four characters 3 (Modifications of PPG morphology), 4 (Branching of the PPG), 9 (Overall structure of the MG), and 13 (Structure of the inner walls of the MG) were recoded (Table S2). This procedure, at least partly, reduced the problem of low numbers of cases per category and is conservative regarding our hypothesis that male beewolves should show pronounced differences in their gland morphology.

According to the results of an initial CATPCA analysis with the combined dataset (Table S2) including all 14 characters of MG and PPG, several changes to the dataset and the analysis parameters were specified according to Linting et al. [5] and Linting and van der Kooij [6]. First, based on the relatively low Eigenvalues (= variance accounted for) obtained for variables 9 (Structure of the MG) and 13 (Structure of the inner walls of the MG) in the initial CATPCA run (Table S3), we decided to exclude these variables from the final analysis [6]. Second, to facilitate the interpretation of the resulting CATPCA plot, we also excluded character 11 (Location of the MG), as the initial analysis revealed a strong redundancy of this character with regard to character 10 (Relative size of the MG) (i.e. same vector coordinates for both characters in the CATPCA plot; see Fig. 4, main text). Despite the somewhat higher Eigenvalue of 11 (Location of the MG, Table S3), we rather retained 10 (Relative size of the MG) in the analysis as we deemed the size of the MG the biologically more relevant trait. Thus, 11 morphological characters were retained in the final CATPCA analysis. Third, while in the initial analysis the level of all variables used for the optimal scaling transformation was nominal, in the final CATPCA run, for characters 2 (Relative size of the PPG), 3 (Branching of the PPG), 10

(Relative size of the MG), and 12 (Branching of the MG) the analysis level was changed to ordinal, based on their transformation plots (i.e. the categories of a variable plotted against the category quantifications assigned to it by the optimal scaling transformation [5, 6]). Moreover, the phylogenetic/ phylogeographic affiliation of the species was included as a supplementary variable in the final CATPCA run. A supplementary variable does not influence the computation of the CATPCA solution, but is rather fitted to the solution afterwards to depict its relation to the other variables in the model [4].

In certain taxonomic groups of the Philanthinae the MG is missing (see main text: "Results"), leading to structural zeros due to nested variables in the dataset (e.g. information on MG size and branching missing due to the absence of this gland). The CATPCA offers an elegant way to handle such structural zeros, by giving the option to assign an extra category to missing values during the transformation process, rather than omit them from the analysis [4, 6]. This option was applied to MG characters.

2.2 Phylogenetic generalized least squares regressions on gland size and complexity

We tested for an opposing trend between MG and PPG with regard to their size and complexity by applying phylogenetic generalized least squares regressions based on the molecular phylogeny of Kaltenpoth et al. [3] in R (Version 3.3.3, [7]) using the package ‘ape’ [8]. As with the CATPCA, *P. albopilosus* (ID 18) was excluded from this analysis, as well as the Nearctic *Philanthus gibbosus* (ID 22), for which the size of the PPG reservoir could not be assessed (Tables S1, S2). Moreover, since the molecular phylogeny comprised only one unidentified *Cerceris* species [3], we used only *C. rybyensis* (ID 2) and *Clypeadon laticinctus* (ID 3, omitting *Cerceris quinquefasciata*, ID 4). We regressed MG and PPG size and complexity, respectively, using phylogenetic independent contrasts by applying the ‘gls’-function of the ‘ape’-package with the correlation argument ‘corBrownian’. The amount of phylogenetic signal in the possible correlations of gland size and complexity was assessed by estimating Pagel’s λ [9] using the ‘gls’-function with the correlation argument ‘corPagel’. Pagel’s λ is an estimate for the strength of phylogenetic signal in the data, with values close to 0 indicating no phylogenetic signal, whereas values close to 1 indicate a strong phylogenetic signal (e.g. [10]). For both, size and complexity the software estimated λ-values close to 1. However, possibly due to the comparatively small number of species in our data set [10], testing these estimated models against models with $\lambda=0$ and $\lambda=1$ (created by using the ‘gls’-function with the correlation argument ‘corPagel’ with ‘value=0’ or ‘value=1’, respectively), by likelihood ratio χ^2 tests (using the ‘anova’-function of ‘ape’), revealed that for both, size and complexity, λ was significantly different from 0 (size and complexity: $p < 0.001$ respectively) and from 1 (size: $p = 0.013$, complexity: $p = 0.0065$). This is

probably the result of the relatively small number of species under study. As a consequence, the results of the correlation analysis with the estimated λ of 1 have to be interpreted with care.

2.3 Hierarchical cluster analysis

The hierarchical cluster analysis (HCA) was based on 13 of the 14 gland characters. Character 8 (Presence of the MG) was excluded, as in the combined dataset it would have been largely redundant with regard to character 10 (Relative size of the MG, Table S2). Fifteen species of the Philanthinae completely lack the MG (see main text: "Results") so that the MG characters could not be defined. To assess a possible effect of these structural zeros on the grouping of species, we repeated the analysis with the most important MG characters combined to one, thus avoiding structural zeros. In this compound character, we summarized the information from the four MG characters 10 (Relative size of MG), 11 (Location of MG within the head capsule), 12 (Branching of MG reservoir), and 14 (Type of gland cells associated with the MG) by assigning a separate category to each of the different combinations of character states in the four original characters (12 different combinations). Yet, we could not include all original MG characters, as this would have resulted in too many categories. We excluded characters 9 (Overall structure of the MG) and 13 (Structure of the inner walls of the MG), based on their comparatively small impact on the clustering of species in the CATPCA (i.e. low Eigenvalues, see section 2.1 of the additional methods above and Table S3). The use of a compound character for the MG altered the grouping of single species (result not shown), but did not change the general outcome as compared to the HCA including structural zeros.

2.4 Phylogenetic trends: Cophylogenetic analysis

The cophylogenetic analysis was run with the default cost parameters of Jane 4, using all possible combinations of either edge- or node-based cost models and either randomizing the parasite (i.e. morphology-based) tree (β=-1) or permuting host-parasite associations (100 resamplings, respectively). After having tested several combinations, which did not influence the results, the number of generations was set to 30 and the population size to 300 for all analyses. As the molecular phylogeny of Kaltenpoth et al. [3] included more species than our morphology-based cluster analysis, we conducted the cophylogenetic analysis using (1) the original molecular phylogeny from Kaltenpoth et al. [3], (2) the molecular phylogeny including only the 33 species also included in our morphology-based tree, and (3) the molecular phylogeny including only the same 33 species, combined with a morphology-based tree in which we treated all species showing no difference in gland morphology (see main text: "Results", Tables S1 and S2) as a single tip using the possibility in Jane 4 to assign multiple host associations between parasite and host trees.

3 Digital photos

All digital microscope photos (main text: Fig.2, Fig. S2) were optimized for color, contrast, and sharpness using the software Adobe Photoshop Elements (Version 5.0; Microsoft, Redmond, USA).

Additional Tables and Figures

Table S1 'Full dataset' of the morphological characters as recorded in the comparative analysis of head gland morphology of male Philanthinae: A total of 14 morphological characters of the postpharyngeal gland (PPG) and the mandibular gland (MG) of 33 species and one subspecies were defined. Species IDs correspond to Table 1 in the main text. The numbering of the characters and the numeric coding of the character states correspond to the description in section 1 above. (?) character state could not be determined, (-) character not present in this species.

		PPG							MG						
**ID**	**Species**	**1**	**2**	**3**	**4**	**5**	**6**	**7**	**8**	**9**	**10**	**11**	**12**	**13**	**14**
**1**	*Cerceris quinquefasciata*	1	0	0	0	-	1	0	1	0	2	3	2	1	0
**2**	*Cerceris rybyensis*	1	1	0	0	-	1	0	1	1	2	2	1	1	3
**3**	*Clypeadon laticinctus*	0	1	2	0	1	1	0	1	0	2	2	1	0	0
**4**	*Philanthinus quattuordecimpunctatus*	0	1	4	1	0	0	0	1	2	2	2	0	1	0
**5**	*Philanthus* cf. *basalis*	1	2	3	1	-	?	0	1	4	2	1	0	1	2
**6**	*Philanthus pulcherrimus*	0	2	5	2	0	0	4	0	-	-	-	-	-	-
**7**	*Philanthus spec* (India)	1	2	5	4	-	0	3	1	2	1	1	0	3	2
**8**	*Philanthus venustus*	0	2	7	4	0	2	2	0	-	-	-	-	-	-
**9**	*Philanthus capensis*	1	2	3	1	-	2	0	1	2	2	1	1	2	2
**10**	*Philanthus coronatus*	1	2	5	3	-	0	3	1	2	1	1	0	2	2
**11**	*Philanthus fuscipennis*	1	2	5	2	-	?	3	1	2	1	1	1	2	2
**12**	*Philanthus histrio*	1	2	5	4	-	2	3	1	2	0	0	0	3	1
**13**	*Philanthus loefflingi*	1	2	5	2	-	2	3	1	2	1	1	1	2	2
**14**	*Philanthus melanderi*	1	2	5	2	-	2	3	1	2	1	1	0	2	2
**15**	*Philanthus rugosus*	1	2	5	4	-	2	3	1	2	0	0	0	3	1
**16**	*Philanthus triangulum triangulum*	1	2	3	1	-	1	0	1	4	2	1	0	4	2
**17**	*Philanthus triangulum diadema*	1	2	3	1	-	1	0	1	4	2	1	0	4	2
**18**	*Philanthus albopilosus*	1	1	0	1	-	0	0	0	-	-	-	-	-	-
**19**	*Philanthus barbiger*	0	2	8	4	0	0	4	0	-	-	-	-	-	-
**20**	*Philanthus bicinctus*	0	2	7	4	0	0	4	0	-	-	-	-	-	-
**21**	*Philanthus crotoniphilus*	0	2	8	4	0	0	4	0	-	-	-	-	-	-
**22**	*Philanthus gibbosus*	?	?	?	?	?	0	4	0	-	-	-	-	-	-
**23**	*Philanthus gloriosus*	0	2	7	2	?	0	4	0	-	-	-	-	-	-
**24**	*Philanthus multimaculatus*	0	2	8	3	0	0	4	1	3	0	0	0	0	1
**25**	*Philanthus occidentalis*	?	2	?	4	?	0	4	0	-	-	-	-	-	-
**26**	*Philanthus pacificus*	0	2	8	4	0	0	4	0	-	-	-	-	-	-
**27**	*Philanthus parkeri*	0	2	8	4	0	0	4	0	-	-	-	-	-	-
**28**	*Philanthus politus*	0	2	8	4	0	0	4	0	-	-	-	-	-	-
**29**	*Philanthus psyche*	0	2	8	4	0	0	4	0	-	-	-	-	-	-
**30**	*Philanthus pulcher*	0	2	8	4	0	0	4	0	-	-	-	-	-	-
**31**	*Philanthus ventilabris*	0	2	?	1	?	0	4	0	-	-	-	-	-	-
**32**	*Trachypus elongatus*	1	2	3	1	-	0	0	1	3	1	0	0	1	1
**33**	*Trachypus flavidus*	1	2	5	2	-	0	1	1	?	0	0	?	0	1
**34**	*Trachypus patagonensis*	?	?	?	?	?	?	0	1	4	1	0	?	0	1

Table S2 'Combined dataset' used for the statistical analyses of head gland morphology of male Philanthinae: For the four characters shaded in grey, some character states were pooled (see section

1 above) as compared to the 'full dataset' (Table S1). Note that *T. patagonensis* (ID 34) has been excluded from this dataset since for most of the characters no reliable information could be obtained (Table S1 and main text: "Data matrix for statistical analysis"). Species IDs correspond to Table 1 in the main text. The numbering of the characters and the numeric coding of the character states correspond to the description in section 1 above. (?) character state could not be determined, (-) character not present in this species.

		PPG							MG						
**ID**	**Species**	**1**	**2**	**3**	**4**	**5**	**6**	**7**	**8**	**9**	**10**	**11**	**12**	**13**	**14**
**1**	***Cerceris quinquefasciata***	1	0	0	0	-	1	0	1	0	2	3	2	1	0
**2**	***Cerceris rybyensis***	1	1	0	0	-	1	0	1	0	2	2	1	1	3
**3**	***Clypeadon laticinctus***	0	1	2	0	1	1	0	1	0	2	2	1	0	0
**4**	***Philanthinus quattuordecimpunctatus***	0	1	4	1	0	0	0	1	0	2	2	0	1	0
**5**	***Philanthus* cf. *basalis***	1	2	3	1	-	?	0	1	2	2	1	0	1	2
**6**	***Philanthus pulcherrimus***	0	2	5	2	0	0	4	0	-	-	-	-	-	-
**7**	***Philanthus spec* (India)**	1	2	5	2	-	0	3	1	0	1	1	0	2	2
**8**	***Philanthus venustus***	0	2	7	2	0	2	2	0	-	-	-	-	-	-
**9**	***Philanthus capensis***	1	2	3	1	-	2	0	1	0	2	1	1	1	2
**10**	***Philanthus coronatus***	1	2	5	2	-	0	3	1	0	1	1	0	1	2
**11**	***Philanthus fuscipennis***	1	2	5	2	-	?	3	1	0	1	1	1	1	2
**12**	***Philanthus histrio***	1	2	5	2	-	2	3	1	0	0	0	0	2	1
**13**	***Philanthus loefflingi***	1	2	5	2	-	2	3	1	0	1	1	1	1	2
**14**	***Philanthus melanderi***	1	2	5	2	-	2	3	1	0	1	1	0	1	2
**15**	***Philanthus rugosus***	1	2	5	2	-	2	3	1	0	0	0	0	2	1
**16**	***Philanthus triangulum triangulum***	1	2	3	1	-	1	0	1	2	2	1	0	2	2
**17**	***Philanthus triangulum diadema***	1	2	3	1	-	1	0	1	2	2	1	0	2	2
**18**	***Philanthus albopilosus***	1	1	0	1	-	0	0	0	-	-	-	-	-	-
**19**	***Philanthus barbiger***	0	2	7	2	0	0	4	0	-	-	-	-	-	-
**20**	***Philanthus bicinctus***	0	2	7	2	0	0	4	0	-	-	-	-	-	-
**21**	***Philanthus crotoniphilus***	0	2	7	2	0	0	4	0	-	-	-	-	-	-
**22**	***Philanthus gibbosus***	?	?	?	?	?	0	4	0	-	-	-	-	-	-
**23**	***Philanthus gloriosus***	0	2	7	2	?	0	4	0	-	-	-	-	-	-
**24**	***Philanthus multimaculatus***	0	2	7	2	0	0	4	1	1	0	0	0	0	1
**25**	***Philanthus occidentalis***	?	2	?	2	?	0	4	0	-	-	-	-	-	-
**26**	***Philanthus pacificus***	0	2	7	2	0	0	4	0	-	-	-	-	-	-
**27**	***Philanthus parkeri***	0	2	7	2	0	0	4	0	-	-	-	-	-	-
**28**	***Philanthus politus***	0	2	7	2	0	0	4	0	-	-	-	-	-	-
**29**	***Philanthus psyche***	0	2	7	2	0	0	4	0	-	-	-	-	-	-
**30**	***Philanthus pulcher***	0	2	7	2	0	0	4	0	-	-	-	-	-	-
**31**	***Philanthus ventilabris***	0	2	?	1	?	0	4	0	-	-	-	-	-	-
**32**	***Trachypus elongatus***	1	2	3	1	-	0	0	1	1	1	0	0	1	1
**33**	***Trachypus flavidus***	1	2	5	2	-	0	1	1	?	0	0	?	0	1

Table S3 Eigenvalues of the 14 morphological characters of the head glands of male Philanthinae included in the initial CATPCA run (Eigenvalue initial run) and the Eigenvalues of the 11 characters retained in the final CATPCA run (Eigenvalue final run). The numbering of the characters (No.) corresponds to the list of characters in the main text, section "Morphological characters" and section 1 above. Characters shaded in grey were excluded from the final CATPCA run (see section 2.1 above).

No.	Character	Eigenvalue initial run	Eigenvalue final run
1	Overall structure of the PPG	1.242	1.087
2	Relative size of the PPG	0.960	0.914
3	Modifications of PPG morphology	1.185	1.008
4	Branching of the PPG	0.986	0.963
5	Number of openings of the lower PPG	1.159	1.006
6	Structure of the inner walls of the PPG	0.987	0.843
7	Type of gland cells associated with the PPG	1.507	1.207
8	Presence of the MG	1.531	1.212
9	Overall structure of the MG	0.305	-
10	Relative size of the MG	0.584	0.614
11	Location of the MG in the head capsule	0.964	-
12	Branching of the MG	0.480	0.459
13	Structure of the inner walls of the MG	0.306	-
14	Type of gland cells associated with the MG	0.944	0.920

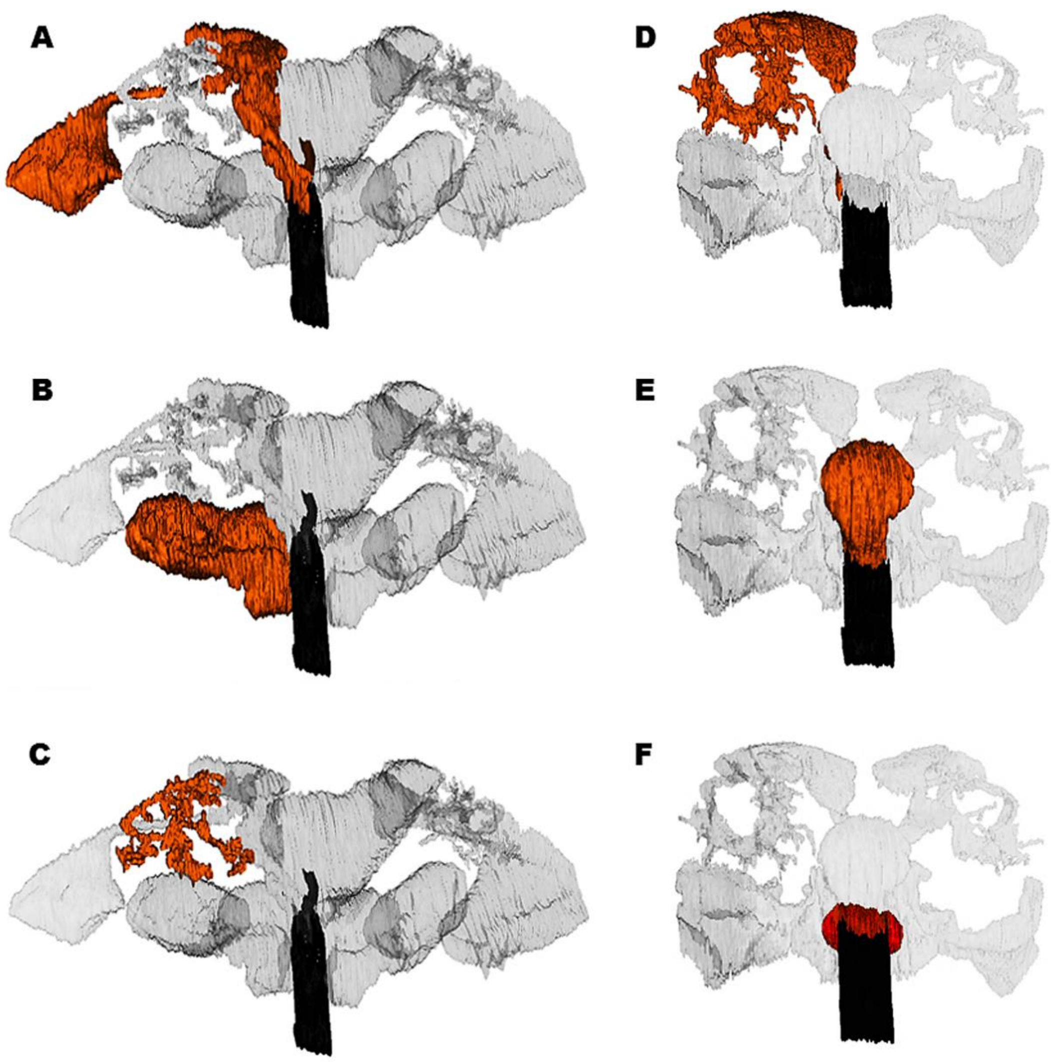

Figure S1 Morphological modifications of the postpharyngeal gland (PPG) of male Philanthinae. The PPG of males of all investigated Philanthinae is made up of different combinations of six distinct morphological elements (see character 3 in section 1 above), which are illustrated here by examples of the PPGs of the Afrotropical *Philanthus rugosus* (A - C) and the Nearctic *Philanthus politus* (D - F) [whole PPG shown in light grey, in each panel the described element is highlighted in either orange (upper PPG) or red (lower PPG, panel F)]. Note that, with the exception of (E) and (F), the described elements are paired structures. **(A)** Unbranched tube-shaped reservoir, directly originating from the pharynx (black) and extending dorsally subjacent to the cuticle of the head capsule (dPPG in Fig. 1, main text); the size of this part can vary considerably among species. **(B)** Unbranched, tube- or sac-shaped reservoir originating from (A) and extending laterally anterior to the brain (aPPG in Fig. 1, main text); also this part of the PPG shows considerable interspecific size variation. **(C)** Fine branches originating from (A) and subsequently ramifying before reaching one or several of the associated secretory cells (not shown) (see also character 7 in section 1 above). **(D)** Gland reservoir originating dorsally from the pharynx and extending posterior around the brain associated with short branches, thus resembling a combination of (A) and (C) (dPPG in Fig. 1, main text); yet, unlike (A) the reservoir

has a very specific coiled appearance and, unlike (C), the fine, comparatively short branches originate in regular intervals and do not further ramify before reaching associated secretory cells (not shown). **(E)** Unpaired, sac-like dorsal evagination at the opening of the PPG to the pharynx. **(F)** Small, unbranched ventral evagination of the pharynx (referred to as ‘lower PPG’ in the main text).

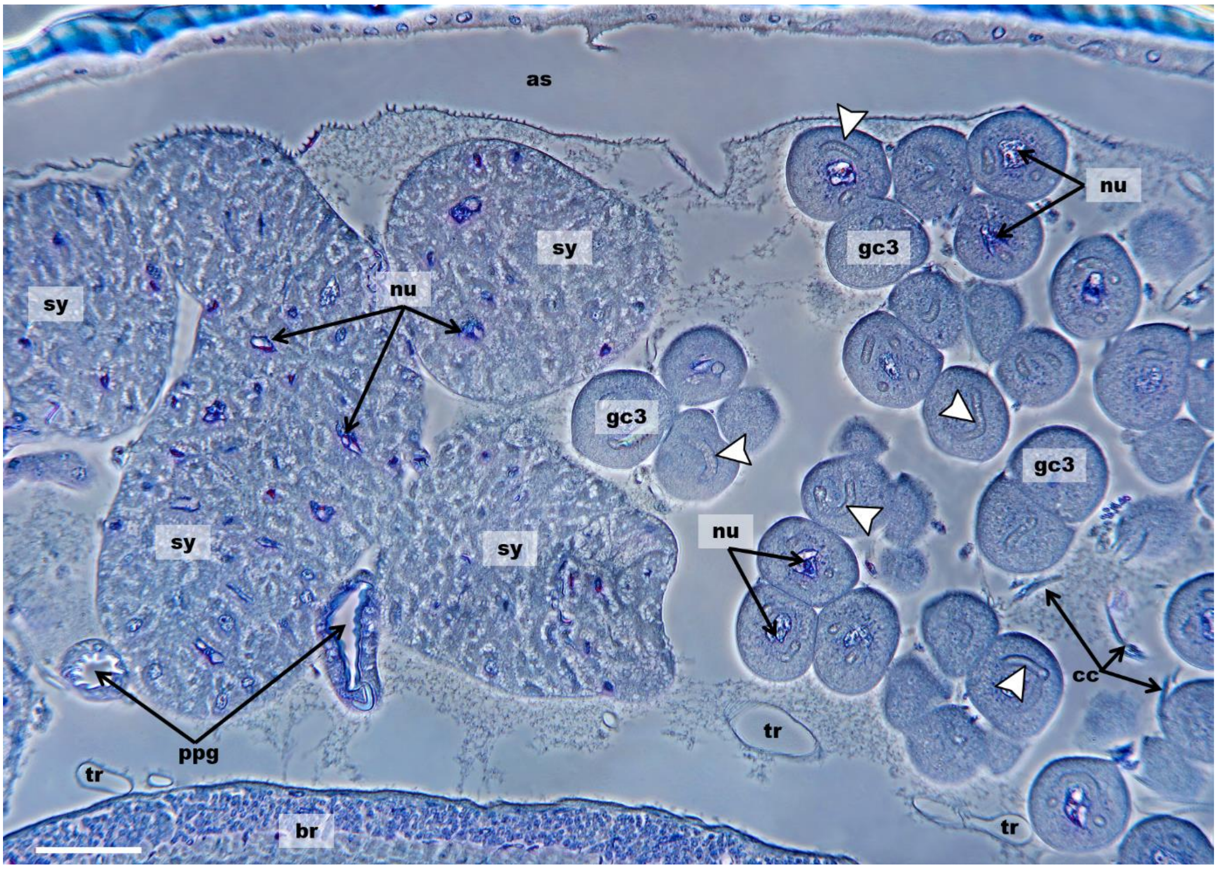

Figure S2 Semithin sagittal section through the head of a male *Philanthus rugosus* showing parts of both, the postpharyngeal gland (PPG) and the mandibular gland (MG), each with associated secretory cells. Multinuclear syncytia surround the fine branches of the PPG reservoir (left side), while the MG is associated with typical NQ-class 3 cells (right side) characterized by end apparatus (white arrow heads) and conducting canals that connect the gland cells with the MG reservoir. Abbreviations: as, air sac; br, brain; cc, conducting canal; gc3, NQ-class 3 gland cells; nu, cell nucleus (with nucleoli); ppg, fine branches of the PPG; sy, multinuclear syncytia; tr, tracheole. Scale bar = 50 µm.

Additional Results

Phylogenetic trends in gland morphology: Hierarchical cluster analysis

The clustering of species in the hierarchical cluster analysis (see main text: section "Phylogenetic trend in gland morphology"), largely, but not entirely, corresponds to the phylogenetic and phylogeographic relationships within the subfamily (according to [3]). In the following, important shifts in in gland morphology are described (Numbers in circles in Fig. S3). (1) All species lacking the MG and/or possessing the most complex type of gland cells associated with the PPG (i.e. multinuclear syncytia or cell aggregations interspersed with small cells), that is all Nearctic *Philanthus* (including *P. multimaculatus* (ID 24) possessing an MG, and *P. albopilosus* (ID 18) completely lacking secretory cells) as well as the Indian *P. pulcherrimus* (ID 6) and the Palearctic *P. venustus* (ID 8), are separated from those philanthine species possessing both MG and PPG but lacking the small cells interspersed in the syncytia of the PPG. (2) Within the cluster possessing both head glands, members of the tribes Cercerini and Aphilanthopsini (IDs 1-3) as well as the one investigated representative of the genus *Philanthinus* (ID 4) form a separate cluster, as their MGs are either much larger than their PPGs or at least of approximately equal volume, while in the other species in this cluster the MG is smaller than the PPG. (3) The two *Trachypus* species are placed into a separate branch, presumably by a unique combination of traits, including the lack of the lower part of the PPG, the presence of only single NQ-class 3 cells at the MG, and the possession of only the lower part of the MG (note however, that the later only holds true for *T. elongatus* (ID 32), while the structure of the MG of *T. flavidus* could not be determined (Table S1 and S2), hence the clustering of *Trachypus* may be interpreted with caution). (4) Within the cluster of Palearctic, Indian, and Afrotropical *Philanthus*, species possessing gland cells associated with both the MG and PPG (IDs 7 and 10-15), are separated from the four species of this cluster possessing only gland cells associated with the MG (IDs 5, 9, 16, 17). (5) The two subspecies of *P. triangulum* (IDs 16 and 17) and the Indian *P.* cf. *basalis* (ID 5) form a separate cluster based on the occurrence of both lower and upper reservoir of the MG in these species. (6) Unlike all other Palearctic/Afrotropical *Philanthus*, *P. histrio* (ID 12) and *P. rugosus* (ID 15) possess only single NQ-class 3 cells and are placed into a separate branch. (7) *P. albopilosus* (ID 18), possessing only a very small PPG and no gland cells, is separated from all other Nearctic *Philanthus*, possessing well developed PPGs. (8) *P. multimaculatus* (ID 24) is placed into an unique branch within the Nearctic *Philanthus* as it is the only species of this cluster possessing an MG. (9) The Palearctic *P. venustus* (ID 8) is placed into an unique branch as the secretory cells of its PPG form aggregations of single cells, rather than multinuclear syncytia.

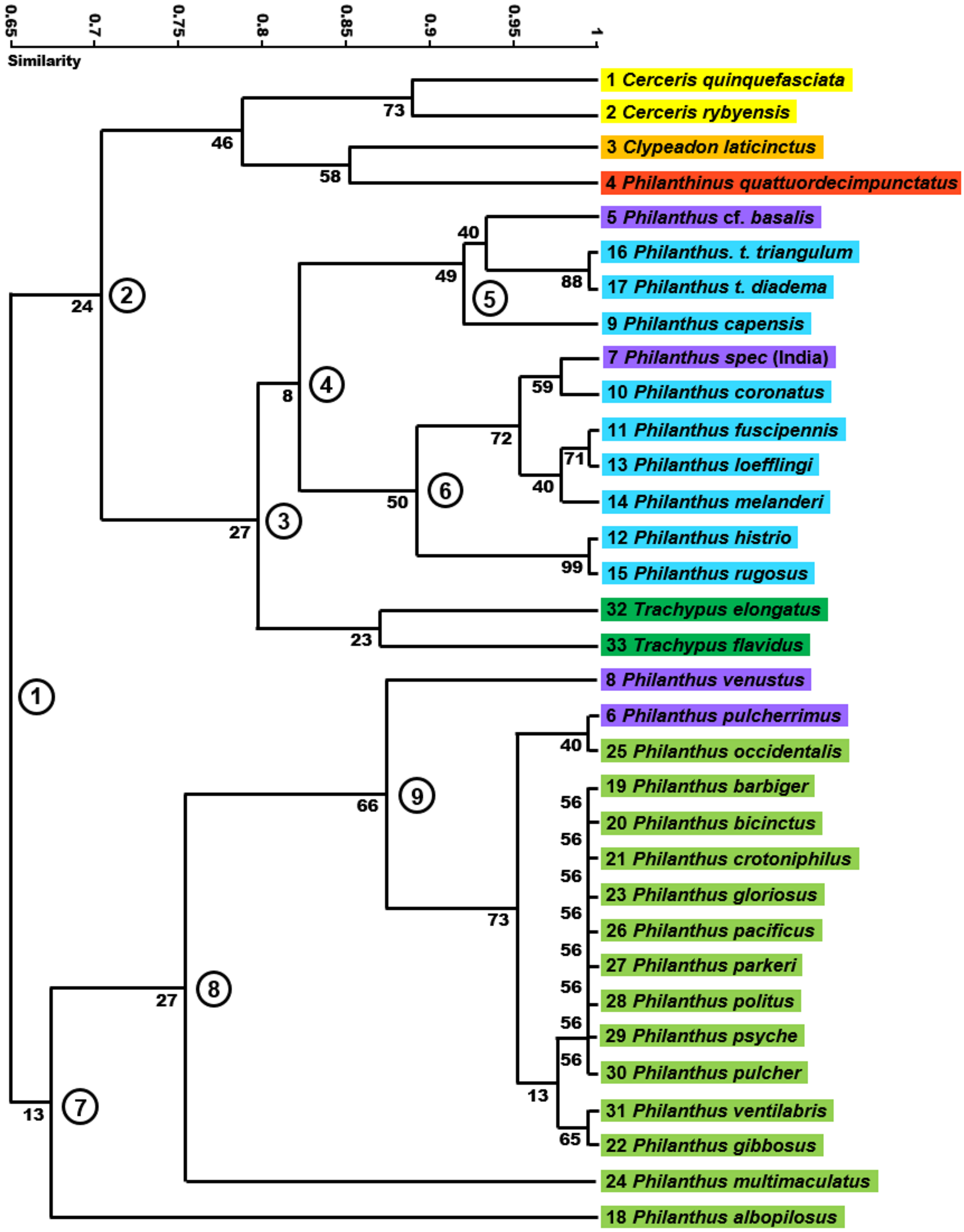

Figure S3 Dendrogram resulting from the hierarchical cluster analysis of head gland morphology of male Philanthinae. Color code: yellow, genus *Cerceris*; orange, genus *Clypeadon*; red, genus *Philanthinus*; purple, Palearctic/Indian *Philanthus*; blue, Palearctic/Afrotropical *Philanthus*; light green, Nearctic *Philanthus*; dark green, genus *Trachypus*. Species IDs correspond to Table 1, main text. Numbers in circles indicate important shifts in gland morphology (see text above for details). Not encircled numbers at nodes are bootstrap values. Bray-Curtis was used as the similarity measure; N bootstrap replicates = 10,000.

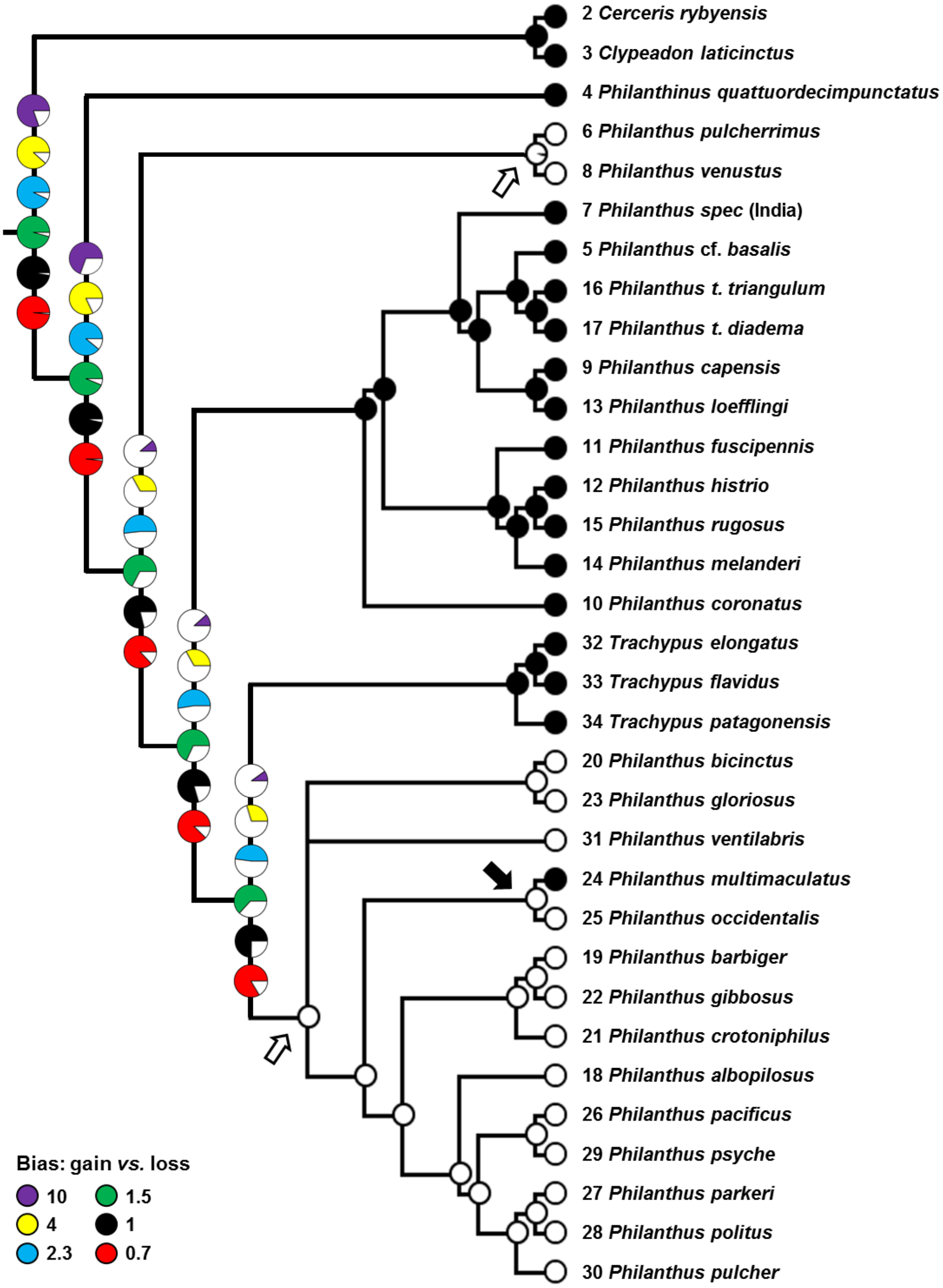

Figure S4 Ancestral state reconstruction for the occurrence of the mandibular gland (MG) of male Philanthinae. The maximum likelihood reconstruction of presence *vs.* absence of the MG (character 8, Tables S1 and S2) was based on the molecular phylogeny of Kaltenpoth et al. [3]. Pie charts at the nodes give the ML probabilities for presence (color) *vs.* absence (white) of an MG. For relevant nodes results of models with different biases are shown (Color code see key in the figure), for all other nodes the results of the unbiased model are given (i.e. gains and losses equally likely, black). The unbiased model revealed two independent losses (white arrows) and one gain (black arrow) of the MG among the investigated species.

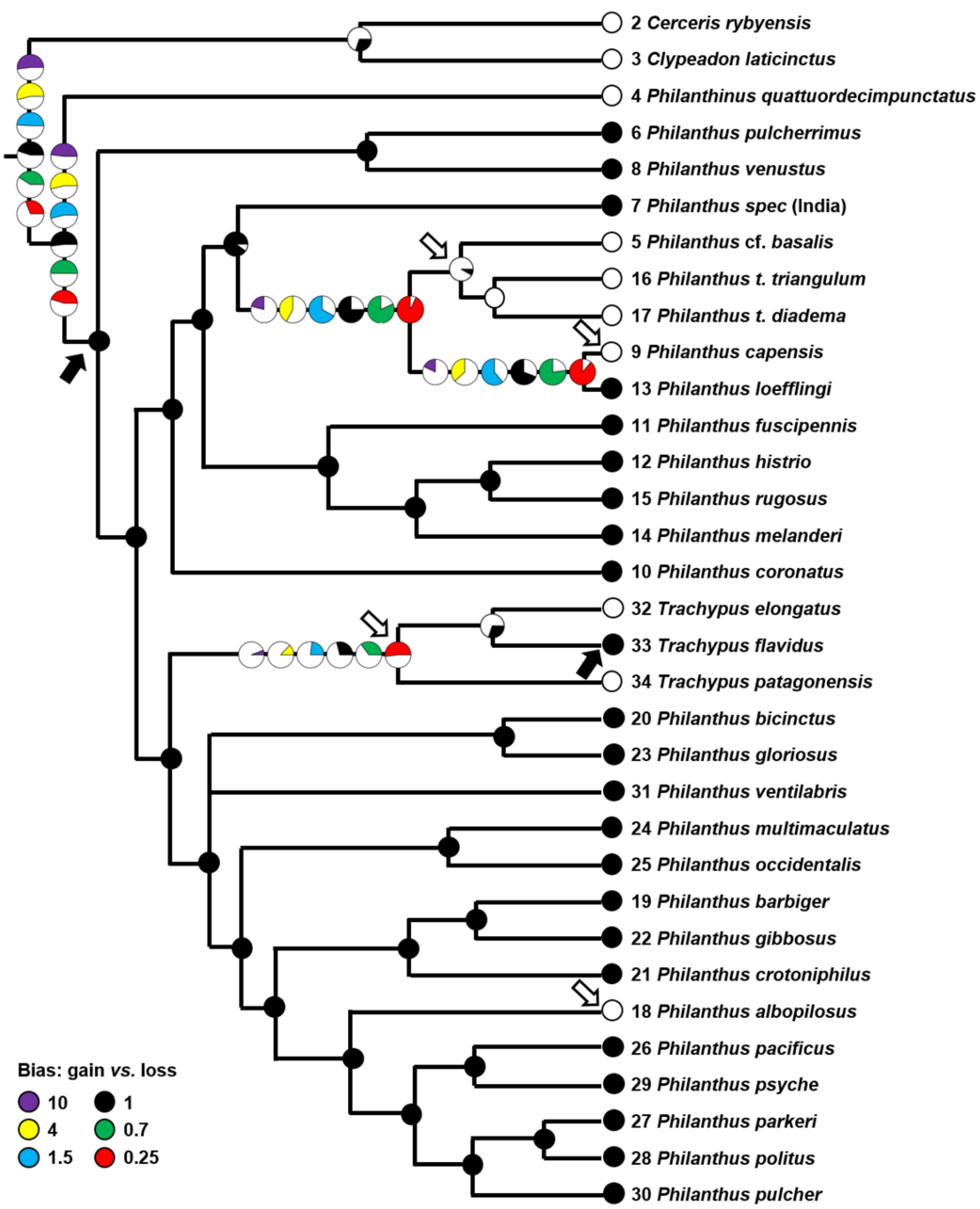

Figure S5 Ancestral state reconstruction for the presence of the presumed secretory cells of the postpharyngeal gland (PPG) in male Philanthinae. The maximum likelihood reconstruction of presence *vs.* absence of the presumed secretory cells (state 0 *vs.* all other states of character 7, Tables S1 and S2) was based on the molecular phylogeny of Kaltenpoth et al. [3]. Pie charts at the nodes give the ML probabilities for the presence (color) *vs.* absence (white) of secretory cells. For relevant nodes results of models with different biases are shown (Color code see key in the figure), for all other nodes the results of the unbiased model are given (i.e. gains and losses equally likely, black). The unbiased model revealed two independent gains (black arrows) and four independent losses (white arrows) of the secretory cells of the PPG among the investigated species.

Additional References

1. Noirot C, Quennedey A. Fine-structure of insect epidermal glands. Annu Rev Entomol. 1974;19:61-80.

2. Alexander BA. A cladistic analysis of the subfamily Philanthinae (Hymenoptera: Sphecidae). Syst Entomol. 1992;17(2):91-108.

3. Kaltenpoth M, Roeser-Mueller K, Koehler S, Peterson A, Nechitaylo TY, Stubblefield JW, Herzner G, Seger J, Strohm E. Partner choice and fidelity stabilize coevolution in a Cretaceous-age defensive symbiosis. Proc Natl Acad Sci U S A. 2014;111(17):6359-6364.

4. Meulman J, van der Kooij A, Heiser WJ. Principal components analysis with nonlinear optimal scaling transformations for ordinal and nominal data. In: Kaplan D, editor. Handbook on quantitative methodology for the social sciences. London: Sage; 2004. p. 49-70.

5. Linting M, Meulman JJ, Groenen PJF, van der Kooij AJ. Nonlinear principal components analysis: introduction and application. Psychol Methods. 2007;12:336-358.

6. Linting M, van der Kooij A. Nonlinear Principal Components Analysis with CATPCA: a Tutorial. J Pers Assess. 2012;94:12-25.

7. R Core Team. R: A language and environment for statistical computing. R Foundation for Statistical Computing. Vienna. 2017. http://www.R-project.org.

8. Paradis E, Claude J, Strimmer K. APE: Analyses of phylogenetics and evolution in R language. Bioinformatics. 2004;20:289-290.

9. Pagel M. Inferring the historical patterns of biological evolution. Nature. 1999;401(6756):877-884.

10. Münkemüller T, Lavergne S, Bzeznik B, Dray S, Jombart T, Schiffers K, Thuiller W. How to measure and test phylogenetic signal. Methods Ecol Evol. 2012;3(4):743-756.
