# Supplementary material for: Sexual selection and the evolution of male pheromone glands in philanthine wasps (Hymenoptera, Crabronidae)
Source: BMC Evol Biol. 2017 Jun 6;17:128. doi: 10.1186/s12862-017-0963-6 (PMC5461632; doi:10.1186/s12862-017-0963-6)
Supplement: Supplementary file 2 — Additional methods for coding of morphological characters for the aggregated analysis of male and female head gland morphology, additional Table S4. showing the aggregated data matrix used for statistical analyses of male and female gland morphology, additional information on the aggregated categorical principal components analysis, including additional Table S5. giving the Eigenvalues of the aggregated morphological characters from the categorical principal components analysis of male and female gland morphology, additional Figure S6. showing the plot of the aggregated categorical principal components analysis, and additional methods on the calculation of Shannon diversity indices for the aggregated characters. (PDF 739 kb) [file 12862_2017_963_MOESM2_ESM.pdf]

Additional File 2

Sexual selection and the evolution of male pheromone glands in philanthine wasps (Hymenoptera, Crabronidae)

Katharina Weiss, Gudrun Herzner, Erhard Strohm*

Evolutionary Ecology Group, Institute of Zoology, University of Regensburg, Universitätsstr. 31, 93053 Regensburg, Germany

* Corresponding author: Erhard Strohm
Universitätsstr. 31
93053 Regensburg
Germany
Phone: +49-941-943-3072
Fax: +49-941-943-3304
Erhard.Strohm@ur.de

Email addresses: Katharina1.Weiss@ur.de (Katharina Weiss)
Gudrun.Herzner@ur.de (Gudrun Herzner)
Erhard.Strohm@ur.de (Erhard Strohm)

1 Definition and coding of aggregated characters of head glands of males and females

In order to test whether the interspecific diversity in the morphology of postpharyngeal gland (PPG) and mandibular gland (MG) is higher in male than in female Philanthinae, the characters and character states of both sexes had to be represented in the same coordinate system by a categorical principal components analysis (CATPCA). To accomplish this, we created an aggregate data matrix by combining and recoding 13 characters of female head glands derived from Weiss et al. [1] and Weiss et al. (*in preparation*) and 14 characters of male head glands derived from the 'combined data' set of this study (Table S2, additional file 1). The resulting aggregated dataset comprises 17 morphological characters of MG and PPG of both sexes (Table S2, additional file 1; see also main text: "Pattern of interspecific variation in gland morphology"). Thirteen of these 17 joint characters represent combinations of the equivalent characters of both sexes (or, in the case of character 12, a combination of one male and two female characters). The different character states of the joint character represent combinations of one to four states of the combined original characters. However, four characters could not be combined since they were unique to males (characters 16 and 17, Table S4) or to females (characters 14 and 15, Table S4) (see section 3 below for details on the treatment of the resulting structural zeroes due to a missing character or lack of information on the character state). Some characters had been much more finely differentiated in females [1] than in males because in the latter the considerable variation in the same character was often so pronounced that fine-graded variations were not coded. Therefore, for some joint characters different original female character states were equaled to the appropriate state as defined for males.

The following description of the aggregated characters and their states provides the number of the joint character and character state in black, as well as the respective original numbers of the characters and states in males [blue, this study] and females [red, [1]]. Hyphens [-] indicate that characters or character states were not defined in the respective original dataset. For more details, see description of male characters in section 1 of the additional file 1 and [1] for females.

MG:

1 **[8]** **[-]** *Presence of the MG reservoir*. Whereas in some Philanthinae, males lack the MG reservoir a MG is present in all females. Hence, the presence of the MG was not originally included as a character in the dataset of female head gland morphology.

(0) **(0)** **(-)** no MG reservoir

(1) **(1)** **(-)** MG reservoir present

2 **[9]** **[9]** *Structure of the MG reservoir*.

(0) **(0)** **(1)** only the upper part of the MG reservoir is present

(1) **(1)** **(0)** only the lower part of the MG reservoir is present

(2) **(2)** **(2)** both upper and lower parts of the MG reservoir are present

3 **[10]** **[10]** *Relative size of the MG reservoir*. In both sexes we assessed the relative size of the MG as its extension in relation to the head capsule.

(0) **(-)** **(0)** very small MG (spanning < 25% of head capsule)

(1) **(0)** **(1)** **(2)** small MG (spanning approximately 25 - 50% of the head capsule)

(2) **(1)** **(-)** medium sized MG (spanning approximately 50 - 100% of the head capsule)

(3) **(2)** **(-)** large MG (spanning 100 - 200% of the head capsule)

4 **[11]** **[-]** *Location of the MG reservoir in the head capsule*. Due to its relatively small size, the MG of female Philanthinae is always located anterior to the brain. Therefore, this character was not originally included in the investigation of female head glands. Here, all females were assigned to category (0). See Additional file 1 character 11 for definition of character states.

(0) **(0)** **(-)** A

(1) **(1)** **(-)** A + D

(2) **(2)** **(-)** A + D + B

(3) **(3)** **(-)** A + D + B + V

5 **[12]** **[11]** *Branching of the MG reservoir*.

(0) **(0)** **(0)** unbranched: the MG consists of paired sac-like evaginations as reservoirs

(1) **(1)** **(1)** low: the MG consists of paired reservoirs, each with a few distinct branches

(2) **(2)** **(-)** high: the MG consists of paired reservoirs, each with many branches

6 **[13]** **[12]** *Structure of the inner walls of the MG reservoir*. The epithelial cells of the MG reservoir can bear cuticular hairs on their inner side, reaching into the lumen of the gland. In females, these hairs generally occur singularly, but in males of some species several hairs can also jointly originate from the top of or laterally from cuticular ridges.

(0) **(0)** **(0)** unstructured

(1) **(1)** **(1)** bearing scattered fine hairs

(2) **(2)** **(2)** bearing many hairs

7 **[14]** **[13]** *Type of gland cells associated with the MG*.

(0) **(-)** **(0)** the MG reservoir is not associated with gland cells

(1) **(0)** **(-)** MG-type 1: single gland cells that show end apparatuses, thus resembling NQ-class 3 cells, but lack the canal cells typically connecting NQ-class 3 cells to a reservoir; instead, these cells are tightly arranged around the MG and are directly associated with the wall of the reservoir

(2) **(1)** **(1)** MG-type 2: single NQ-class 3 gland cells are connected to the MG reservoir via conducting canals

(3) **(2)** **(-)** MG-type 3: acini, i.e. several NQ-class 3 cells are tightly arranged in cell clusters and jointly connect to the MG reservoir via a bundle of conducting canals

(4) **(3)** **(-)** MG-type 4: different parts of the MG are associated with MG-type 1 and MG-type 2 cells, respectively

PPG:

8 **[1]** **[1]** *Overall structure of the PPG reservoir*.

(0) **(0)** **(0)** PPG consists of both an upper reservoir originating from the dorsal side of the pharynx and a lower evagination originating ventrally from the pharynx

(1) **(1)** **(1)** PPG consists of only the upper reservoir

9 **[-]** **[4]** *Number of openings of the upper part of the PPG to the pharynx.* Female Philanthinae have either a single opening or separate openings for the right and left part of the upper PPG reservoir whereas males have invariably separate openings. Hence, this character was not included in the original dataset of males. For the joint dataset, males were assigned to category (1).

(0) **(-)** **(0)** right and left part of the upper PPG reservoir share one opening to the pharynx

(1) **(-)** **(1)** both right and left part of the upper PPG each have a separate opening to the pharynx

10 [5] [8] *Number of openings of the lower part of the PPG to the pharynx.*

(0) (0) (0) the lower PPG reservoir has one single opening to the pharynx

(1) (1) (1) the lower PPG reservoir has two separate openings to the pharynx

11 [6] [6] *Structure of the inner walls of the PPG*. The variations in density and distribution of epithelial hairs in females [1] are small compared to the respective variation in males. Thus, here we combined all characters states of the female data set.

(0) (0) (0) unstructured

(1) (1) (-) cuticular folding

(2) (2) (1) (2) (3) (4) hairs

12 [3] [2] [7] *Modifications of the PPG morphology*. Since the PPG of female Philanthinae is less complex and shows much less interspecific variation, it was not originally recorded as in males (Table S2, additional file 1). Here, we recoded the two characters that were used to define the shape of the upper and lower PPG of females [1] to match the definitions of PPG 'modules' established for males. We added two new modules that describe the PPG parts of females: **(F)** Unbranched tube-shaped reservoir originating ventrally from the pharynx. **(G)** glove-shaped upper PPG, extending laterally towards the compound eyes in front of the brain and consisting of a common root from which numerous 'fingers' branch off; this part constitutes the typical upper PPG of female Philanthini and is not found in males. See additional file 1 character 3 for definition of character states.

(0) (0) (-) A

(1) (1) (-) A + D

(2) (2) (-) A + E

(3) (-) (0)[1] + (1)[2] A + F

(4) (3) (-) A + B

(5) (4) (-) A + B + D + E

(6) (5) (-) A + B + C

(7) (6) (-) A + B + C + E

(8) (7) (8) (-) A + B + C + D + E

(9) (-) (1)[1] (2)[1] (3)[1] G

(10) (-) (1)[1] (2)[1] (3)[1] + (0)[2] G + E

[1] Character states of female character (2) 'Shape of the upper PPG' of [1]

[2] Character states of female character (7) 'Shape of the lower PPG' of [1]

13 **[7]** **[-]** *Type of gland cells associated with the PPG*. In females the PPG has never been found to be associated with secretory cells, hence this character was not originally defined in the morphological investigation of females. In the 'joint dataset', females are assigned to category (0).

(0) **(0)** **(-)** no gland cells

(1) **(1)** **(-)** PPG-type 1: several gland cells are tightly associated and form cell aggregations that are directly associated with very fine branches of the PPG (see character 17)

(2) **(2)** **(-)** PPG-type 2: similar to PPG-type 1, but the cell aggregations are interspersed with small rounded cells (Fig. 2 D, main text)

(3) **(3)** **(-)** PPG-type 3: the secretory cells consist of syncytia (i.e. aggregations of cells but no cell membranes visible between cells) that are directly associated with very fine branches of the PPG (see character 17) (Fig. 2 E, main text)

(4) **(4)** **(-)** PPG-type 4: similar to PPG-type 3 but the syncytia are interspersed with small rounded cells (similar to those described for PPG-type 2) (Fig. 2 F, main text)

14 **[-]** **[5]** *Relative lateral extension of the upper PPG of females*. For females, the shape of the PPG required that its relative size was estimated as the lateral extension within the head capsule (which represents, however, the longitudinal axis of the gland) as opposed to males where the longitudinal extension was determined (see character 16).

(0) **(0)** 45 % or less

(1) **(1)** 46 - 50 %

(2) **(2)** 51 - 55 %

(3) **(3)** 56 - 60 %

(4) **(4)** 61 - 65 %

(5) **(5)** 66 - 70 %

(6) **(6)** 71 - 75 %

(7) **(7)** > 75 %

15 **[-]** **[3]** *Number of lobes (per side) of the upper PPG of females*. Again, due to the difference in shape of PPG between the sexes, the degree of branching, i.e. the number of 'fingers' of the glove- or comb-shaped female PPGs, was determined by assessing the average number of 'fingers' on each side of the laterally symmetric upper PPG.

(0) **(0)** 1

(1) **(1)** < 10

(2) **(2)** 10 - 15

(3) **(3)** > 15

16 [2] [-] *Relative dorsal extension of the upper PPG reservoir of males*.

(0) (0) small PPG (spanning approximately 25 - 50% of the head capsule)

(1) (1) medium sized PPG (spanning approximately 50 - 100% of the head capsule)

(2) (2) large PPG (spanning 100 - 200% of the head capsule, i.e. it reaches around the brain and extends downwards towards the mandible base ventrally to the brain)

17 [4] [-] *Branching of the PPG reservoir of males*.

(0) (0) unbranched

(1) (1) some quite voluminous branches originating from the main reservoir of the upper PPG

(2) (2) fine branches associated with single gland cell units

2 Aggregated dataset of head gland morphology for both sexes of Philanthinae

Table S4 Aggregated dataset of head gland morphology for both sexes of Philanthinae. Species IDs for males correspond to Table 1, main text. The numbering of the characters and the numeric coding of the character states correspond to the description in section 1 above. (?) character state could not be determined, (-) character not present in this species.

		MG							PPG									
**ID**	**Males**	**1**	**2**	**3**	**4**	**5**	**6**	**7**	**8**	**9**	**10**	**11**	**12**	**13**	**14**	**15**	**16**	**17**
1	*Cerceris quinquefasciata*	1	0	3	3	2	1	1	1	1	-	1	0	0	-	-	0	0
2	*Cerceris rybyensis*	1	0	3	2	1	1	4	1	1	-	1	0	0	-	-	1	0
3	*Clypeadon laticinctus*	1	0	3	2	1	0	1	0	1	1	1	2	0	-	-	1	0
4	*P. quattuordecimpunctatus*	1	0	3	2	0	1	1	0	1	0	0	5	0	-	-	1	1
5	*Philanthus* cf. *basalis*	1	2	3	1	0	1	3	1	1	-	?	4	0	-	-	2	1
6	*Philanthus pulcherrimus*	0	-	-	-	-	-	-	0	1	0	0	6	4	-	-	2	2
7	*Philanthus spec* (India)	1	0	2	1	0	2	3	1	1	-	0	6	3	-	-	2	2
8	*Philanthus venustus*	0	-	-	-	-	-	-	0	1	0	2	8	2	-	-	2	2
9	*Philanthus capensis*	1	0	3	1	1	1	3	1	1	-	2	4	0	-	-	2	1
10	*Philanthus coronatus*	1	0	2	1	0	1	3	1	1	-	0	6	3	-	-	2	2
11	*Philanthus fuscipennis*	1	0	2	1	1	1	3	1	1	-	?	6	3	-	-	2	2
12	*Philanthus histrio*	1	0	1	0	0	2	2	1	1	-	2	6	3	-	-	2	2
13	*Philanthus loefflingi*	1	0	2	1	1	1	3	1	1	-	2	6	3	-	-	2	2
14	*Philanthus melanderi*	1	0	2	1	0	1	3	1	1	-	2	6	3	-	-	2	2
15	*Philanthus rugosus*	1	0	1	0	0	2	2	1	1	-	2	6	3	-	-	2	2
16	*Philanthus t. triangulum*	1	2	3	1	0	2	3	1	1	-	1	4	0	-	-	2	1
17	*Philanthus t. diadema*	1	2	3	1	0	2	3	1	1	-	1	4	0	-	-	2	1
19	*Philanthus barbiger*	0	-	-	-	-	-	-	0	1	0	0	8	4	-	-	2	2
20	*Philanthus bicinctus*	0	-	-	-	-	-	-	0	1	0	0	8	4	-	-	2	2
21	*Philanthus crotoniphilus*	0	-	-	-	-	-	-	0	1	0	0	8	4	-	-	2	2
22	*Philanthus gibbosus*	0	-	-	-	-	-	-	?	1	?	0	?	4	-	-	?	?
23	*Philanthus gloriosus*	0	-	-	-	-	-	-	0	?	?	0	8	4	-	-	2	2
24	*Philanthus multimaculatus*	1	1	1	0	0	0	2	0	1	0	0	8	4	-	-	2	2
25	*Philanthus occidentalis*	0	-	-	-	-	-	-	?	?	?	0	?	4	-	-	2	2
26	*Philanthus pacificus*	0	-	-	-	-	-	-	0	1	0	0	8	4	-	-	2	2
27	*Philanthus parkeri*	0	-	-	-	-	-	-	0	1	0	0	8	4	-	-	2	2
28	*Philanthus politus*	0	-	-	-	-	-	-	0	1	0	0	8	4	-	-	2	2
29	*Philanthus psyche*	0	-	-	-	-	-	-	0	1	0	0	8	4	-	-	2	2
30	*Philanthus pulcher*	0	-	-	-	-	-	-	0	1	0	0	8	4	-	-	2	2
31	*Philanthus ventilabris*	0	-	-	-	-	-	-	0	1	?	0	?	4	-	-	2	1
32	*Trachypus elongatus*	1	1	2	0	0	1	2	1	1	-	0	4	0	-	-	2	1
33	*Trachypus flavidus*	1	?	1	0	?	0	2	1	?	-	0	6	1	-	-	2	2

		MG							PPG									
**ID**	**Females**	**1**	**2**	**3**	**4**	**5**	**6**	**7**	**8**	**9**	**10**	**11**	**12**	**13**	**14**	**15**	**16**	**17**
34	*Cerceris arenaria*	1	0	1	0	0	0	2	0	1	1	2	3	0	3	0	-	-
35	*Cerceris quinquefasciata*	1	0	1	0	0	0	2	0	1	1	2	3	0	0	0	-	-
36	*Cerceris rybyensis*	1	0	0	0	0	0	0	0	1	1	4	3	0	?	0	-	-
37	*Cerceris sabulosa*	1	0	0	0	0	0	0	0	1	1	3	3	0	1	0	-	-
38	*Aphilanthops frigidus*	1	0	1	0	0	0	0	0	0	1	2	3	0	4	0	-	-
39	*Clypeadon laticinctus*	1	0	1	0	0	0	0	0	1	1	2	3	0	2	0	-	-

40	*P. quattuordecimpunctatus*	1	0	1	0	0	0	0	0	0	0	0	10	0	4	1	-	-
41	*Philanthus venustus*	1	0	1	0	0	0	0	0	0	0	2	10	0	4	2	-	-
42	*Philanthus capensis*	1	0	1	0	0	0	0	0	1	0	2	10	0	5	2	-	-
43	*Philanthus coronatus*	1	0	0	0	0	0	2	0	1	0	2	10	0	7	2	-	-
44	*Philanthus loefflingi*	1	0	1	0	0	0	0	0	1	0	2	10	0	6	2	-	-
45	*Philanthus melanderi*	1	0	1	0	0	0	2	0	1	0	2	10	0	3	2	-	-
46	*Philanthus rugosus*	1	0	1	0	0	0	0	0	1	0	2	10	0	4	2	-	-
47	*Philanthus t. triangulum*	1	0	1	0	0	0	2	0	1	0	2	10	0	?	?	-	-
48	*Philanthus t. diadema*	1	0	1	0	0	0	2	0	1	0	2	10	0	5	2	-	-
49	*Philanthus albopilosus*	1	0	0	0	0	0	2	0	1	0	2	10	0	5	2	-	-
50	*Philanthus barbiger*	1	0	1	0	0	0	0	0	0	0	2	10	0	5	2	-	-
51	*Philanthus bicinctus*	1	0	0	0	0	0	0	0	1	0	?	10	0	?	3	-	-
52	*Philanthus crabroniformis*	1	0	1	0	0	0	0	0	?	?	?	?	0	?	?	-	-
53	*Philanthus gibbosus*	1	0	0	0	0	0	2	0	1	0	2	10	0	1	1	-	-
54	*Philanthus multimaculatus*	1	0	1	0	0	0	0	0	0	0	0	10	0	?	2	-	-
55	*Philanthus psyche*	1	0	0	0	0	0	0	0	?	0	2	10	0	?	3	-	-
56	*Philanthus pulcher*	1	0	?	0	?	0	0	0	1	0	2	?	0	?	?	-	-
57	*Philanthus ventilabris*	1	0	1	0	0	0	0	0	0	0	2	10	0	4	2	-	-
58	*Trachypus boharti*	1	0	1	0	0	0	2	1	1	-	2	9	0	7	2	-	-
59	*Trachypus elongatus*	1	0	1	0	0	0	0	0	0	0	2	10	0	6	2	-	-
60	*Trachypus flavidus*	1	?	?	0	?	?	2	1	1	-	2	9	0	6	2	-	-
61	*Trachypus patagonensis*	1	0	1	0	0	0	0	0	?	?	?	?	0	?	?	-	-

3 Aggregated categorical principal components analysis of males and females

The aggregated dataset (Table S4) was subjected to a categorical principal components analysis as described for the data of males (main text: "Categorical principal components analysis" and section 2.1 of the additional file 1) to represent the head gland characters for males and females in the same coordinate system. Based on the results obtained from the initial CATPCA run including all 17 aggregated characters, the following adjustments of the analysis parameters were made: First, based on their relatively low Eigenvalues (= variance accounted for) in the initial CATPCA run, we decided to exclude the three characters (2) 'Structure of the MG', (9) 'Number of openings of the upper PPG to the pharynx', and (10) 'Number of openings of the lower PPG to the pharynx' from the final analysis (Table S5). Second, to facilitate the interpretation of the resulting CATPCA plot, we also excluded character (4) 'Location of the MG in the head capsule', as the initial analysis revealed a strong redundancy of this character with regard to three other characters (i.e. similar vector coordinates of the characters in the CATPCA plot), namely (3) 'Relative size of the MG', (6) ' Structure of the inner walls of the MG', and (7) 'Type of gland cells associated with the MG'. The final CATPCA thus included 13 morphological characters. Third, while the initial analysis level of all variables, i.e. the scaling level used for the optimal scaling transformation, was changed from nominal to ordinal in the final CATPCA run for six characters (Table S5).

Two of the characters that were only defined for one sex included measures of PPG size, i.e. the lateral extension of the females' PPG assessed by the number of semithin sagittal sections of the head capsule containing structures assigned to the PPG reservoir (Character 14) and the dorsal extension of the males' PPG relative to the head capsule (Character 16), respectively. Although both represent PPG size in relation to the head capsule, we did not combine them to a joint character since the approaches to measure lateral and dorsal extension of the differently structured PPGs females of and males, respectively, were too different ([1], this study). Yet, to exclude at least major effects of these separated characters on the grouping of species in the CATPCA, we tentatively combined the two size characters by including the female size categories into the more grossly defined male categories (compare descriptions of characters 14 and 16 in section 1 above). This did not change the results as compared to the analysis including separated characters for male and female PPG size. Thus, we decided to retain the separated characters in the CATPCA.

Table S5 Eigenvalues of the 17 aggregated morphological characters of head gland morphology of male and female Philanthinae included in the initial CATPCA run (Eigenvalue initial run) and the Eigenvalues of the 13 characters retained in the final CATPCA run (Eigenvalue final run). The numbering of the characters (No.) corresponds to the list of characters in section 1 above. Characters shaded in grey were excluded from the final CATPCA run. The optimal scaling transformation (Level) was changed to ordinal (o) in the final CATPCA run, while it was left nominal (n) in the remaining seven characters.

No.	Character	Eigenvalue initial run	Eigenvalue final run	Level
1	Presence of the MG	1.622	1.613	n
2	Structure of the MG	0.204	-	-
3	Relative size of the MG	1.067	1.057	o
4	Location of the MG in the head capsule	1.055	-	-
5	Branching of the MG	0.475	0.483	o
6	Structure of the inner walls of the MG	1.047	1.078	n
7	Type of gland cells associated with the MG	1.112	1.079	n
8	Overall structure of the PPG	0.916	0.940	n
9	Number of openings of the upper PPG to the pharynx	0.310	-	-
10	Number of openings of the lower PPG to the pharynx	0.336	-	-
11	Structure of the inner walls of the PPG	1.211	1.158	n
12	Modifications of the PPG morphology	1.367	1.321	n
13	Type of gland cells associated with the PPG	1.868	1.738	n
14	Relative lateral extension of the upper PPG in females	0.413	0.454	o
15	Number of lobes of the upper PPG in females	0.469	0.533	o
16	Relative dorsal extension of the upper PPG in males	0.849	0.752	o
17	Branching of the PPG reservoir in males	0.923	0.829	o

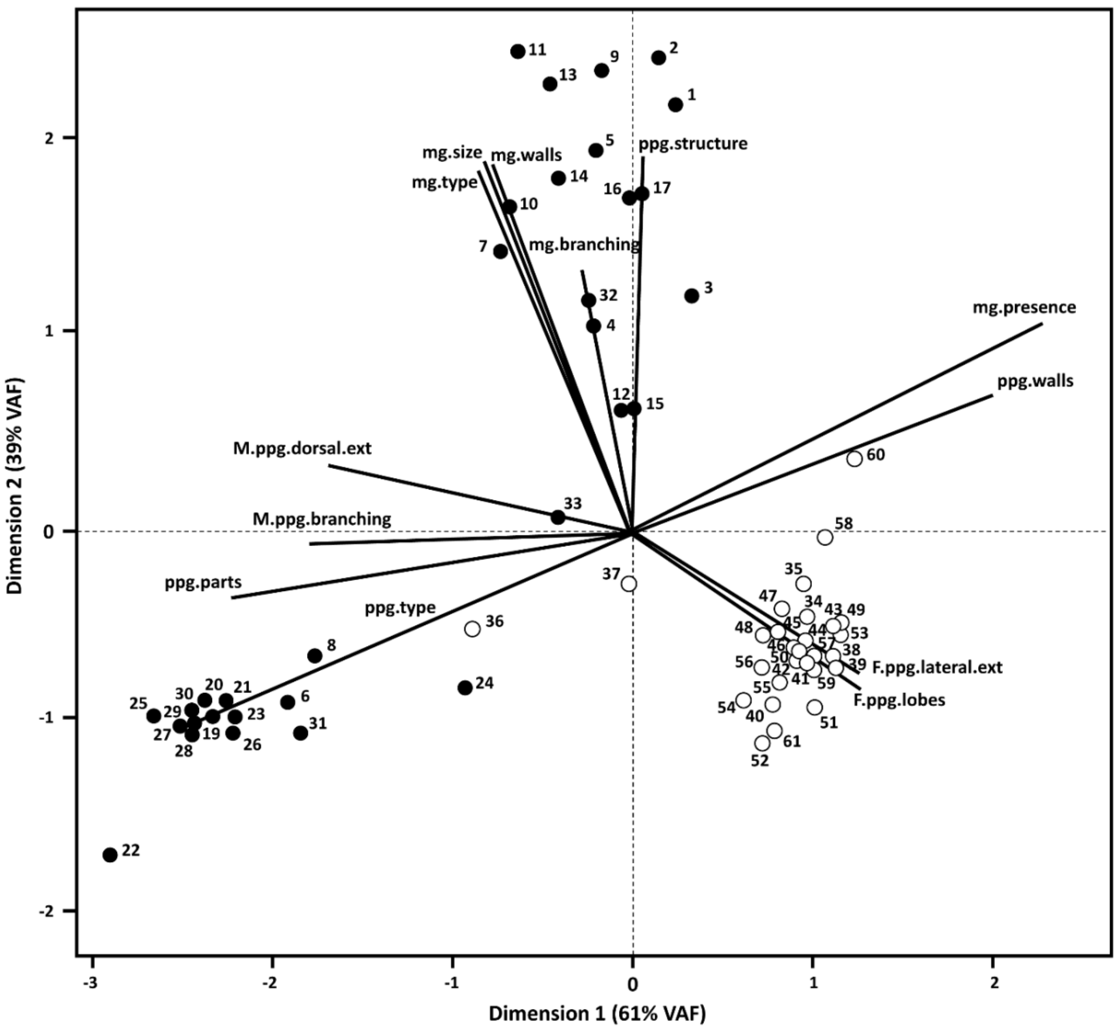

Figure S6 First two dimensions (VAF: percent of variance accounted for) of the categorical principal components analysis (CATPCA) based on the aggregate matrix of morphological characters of the head glands of male (filled circles; IDs correspond to Table 1, main text) and female (open circles) Philanthinae (supported by the maximum possible total Cronbach's α of 1, [2]). While most female data points are closely clustered, the data points for males are much more scattered and show two main aggregations similar to the CATPCA including only males (see Figure 4, main text). Vectors: component loadings of morphological characters. Abbreviations of morphological characters: (mg.branching) branching of the MG, (mg.presence) presence of the MG, (mg.size) size of the MG relative to the head capsule, (mg.type) type of gland cells associated with the MG, (mg.walls) structure of the inner walls of the MG, (ppg.parts) modifications of PPG morphology, (ppg.structure) overall structure of the PPG, (ppg.type) type of gland cells associated with the PPG, (ppg.wall) structure of the inner walls of the PPG, (F.ppg.lateral.ext) lateral extension of the upper PPG relative to the head capsule in females, (F.ppg.lobes) number of lobes of the upper PPG in females, (M.ppg.branching) branching of the PPG reservoir in males (M.ppg.dorsal.ext), dorsal extension of the upper PPG relative to the head capsule in males.

4 Shannon diversity indices for male and female gland morphology

To compare the diversity of gland morphology between the sexes the aggregated dataset was reduced to those characters that were shared by males and females. Therefore, two characters that were only defined in males and two characters that were only defined in females had to be omitted. Following a method described by Tesfaye et al. [3], Shannon diversity indices of the resulting nine characters for males and females were calculated for each joint character for males and females separately, according to the formula:

$$H = -\sum_i \frac{n_i}{n} ln \frac{n_i}{n}$$

With i being the number of categories of a given character, n being the total number of species for which this characters could be assessed, and n_i being the number of species in a given category. The resulting nine indices for both sexes were compared using a Wilcoxon paired test in PAST (Version 2.08b, [4]).

Additional References

1. Weiss K, Strohm E, Kaltenpoth M, Herzner G. Comparative morphology of the postpharyngeal gland in the Philanthinae (Hymenoptera, Crabronidae) and the evolution of an antimicrobial brood protection mechanism. BMC Evol Biol. 2015;15.

2. Heiser W, Meulman J. Homogeneity analysis: exploring the distribution of variables and their nonlinear relationships. In: Greenacre JB, Blasius J, editors. Correspondence analysis in the social sciences: recent developments and applications. 1st ed. New York: Academic Press; 1994. p. 179-209.

3. Tesfaye T, Getachew B, Worede M: Morphological diversity in tetraploid wheat landrace populations from the central highlands of Ethiopia. Hereditas 1991, 114(2):171-176.

4. Hammer Ø, Harper DAT, Ryan PD. PAST: Paleontological statistics software package for education and data analysis. Paleontol Electron. 2001;4.
